# Single-cell analysis of cerebrospinal fluid reveals common features of neuroinflammation

**DOI:** 10.1016/j.xcrm.2024.101733

**Published:** 2024-12-20

**Authors:** Benjamin M. Jacobs, Christiane Gasperi, Sudhakar Reddy Kalluri, Raghda Al-Najjar, Mollie O. McKeon, Jonathan Else, Albert Pukaj, Friederike Held, Stephen Sawcer, Maria Ban, Bernhard Hemmer

**Affiliations:** 1 Department of Clinical Neurosciences, University of Cambridge, Cambridge, UK; 2 Wolfson Institute of Population Health, Queen Mary University of London, London, UK; 3 Department of Neurology, Technical University of Munich, Munich, Germany; 4 Munich Cluster for Systems Neurology (SyNergy), Munich, Germany

**Keywords:** multiple sclerosis, single-cell sequencing, clonal expansion, oligoclonal bands, neuroinflammation, cerebrospinal fluid, omics

## Abstract

Neuroinflammation is often characterized by immune cell infiltrates in the cerebrospinal fluid (CSF). Here, we apply single-cell RNA sequencing to explore the functional characteristics of these cells in patients with various inflammatory, infectious, and non-inflammatory neurological disorders. We show that CSF is distinct from the peripheral blood in terms of both cellular composition and gene expression. We report that the cellular and transcriptional landscape of CSF is altered in neuroinflammation but is strikingly similar across different neuroinflammatory disorders. We find clonal expansion of CSF lymphocytes in all disorders but most pronounced in inflammatory diseases, and we functionally characterize the transcriptional features of these cells. Finally, we explore the genetic control of gene expression in CSF lymphocytes. Our results highlight the common features of immune cells in the CSF compartment across diverse neurological diseases and may help to identify new targets for drug development or repurposing in multiple sclerosis (MS).

## Highlights

• CSF is enriched with B cells and antibody-secreting cells in neuroinflammation
• Immune cells in CSF express a compartment-specific transcriptional program
• *SUB1* expression is increased in B cell clones of MS and neuro-infections
• MS risk alleles—e.g., in *EAF2*—alter gene expression in CSF lymphocytes

## Teaser

Jacobs, Gasperi et al. probe the immune landscape of cerebrospinal fluid in multiple sclerosis and other neuroinflammatory disorders. By combining single-cell gene expression with lymphocyte receptor sequencing, they describe the cellular, transcriptional, and clonal changes that accompany the intrathecal immune response, highlighting the striking similarity across diverse neuroinflammatory conditions.

## Introduction

One of the core features of the vertebrate adaptive immune response is the rapid clonal proliferation of specific lymphocytes on encountering antigen.^1^ This process is vital to efficiently control infections and malignancy but, when aberrantly activated or inadequately regulated, can result in autoimmune disease.^2^^,^^3^^,^^4^ The cerebrospinal fluid (CSF), historically considered an immunologically privileged compartment,^5^ becomes populated with clonally expanded lymphocytes in both healthy aging and in the context of neurological diseases.^6^^,^^7^^,^^8^ In multiple sclerosis (MS) and other neuroinflammatory disorders, clonal expansion of B cells within the CSF produces a limited repertoire of antibodies, which can be detected as oligoclonal bands.^9^^,^^10^ However, it remains unclear how B and T lymphocytes gain access to the CSF in the context of inflammation, what conditions promote clonal expansion, and to what extent these conditions are specific to MS or common to neuroinflammatory states. Understanding these processes may help to shed light on the pathobiology of MS and suggest rational targets for therapeutic intervention.

We therefore sought to characterize the immune landscape of the CSF in non-inflammatory and inflammatory neurological disease states at single-cell resolution. We performed large-scale single-cell sequencing of the CSF and peripheral blood mononuclear cells (PBMCs) in a range of neurological conditions. Our work provides insights into the biology of CSF immune responses in health and disease, building on the insights from earlier efforts based on bulk RNA sequencing, lymphocyte repertoire sequencing, and single-cell sequencing in smaller cohorts.^2^^,^^6^^,^^11^^,^^12^^,^^13^^,^^14^^,^^15^^,^^16^ We demonstrate that immune cells in the CSF exhibit particular features that discriminate them from peripheral blood immune cells, with the majority of these salient features shared across neuroinflammatory states. We show that clonal expansion of CSF B and T cells is observed across different neurological diseases but is most prominent in inflammatory diseases such as MS. Probing the gene expression profiles of clonally expanded lymphocytes revealed common drivers of clonal expansion across inflammatory disorders, suggesting shared mechanisms of aberrant clonal expansion. This study represents the largest single-cell dissection of the intrathecal immune response to date and argues that neuroinflammatory disorders are distinguished by rather subtle quantitative differences in the intrathecal immune milieu rather than stark qualitative differences.

## Results

### CSF is enriched with antibody-secreting cells in neuroinflammation

**Figure 1:**
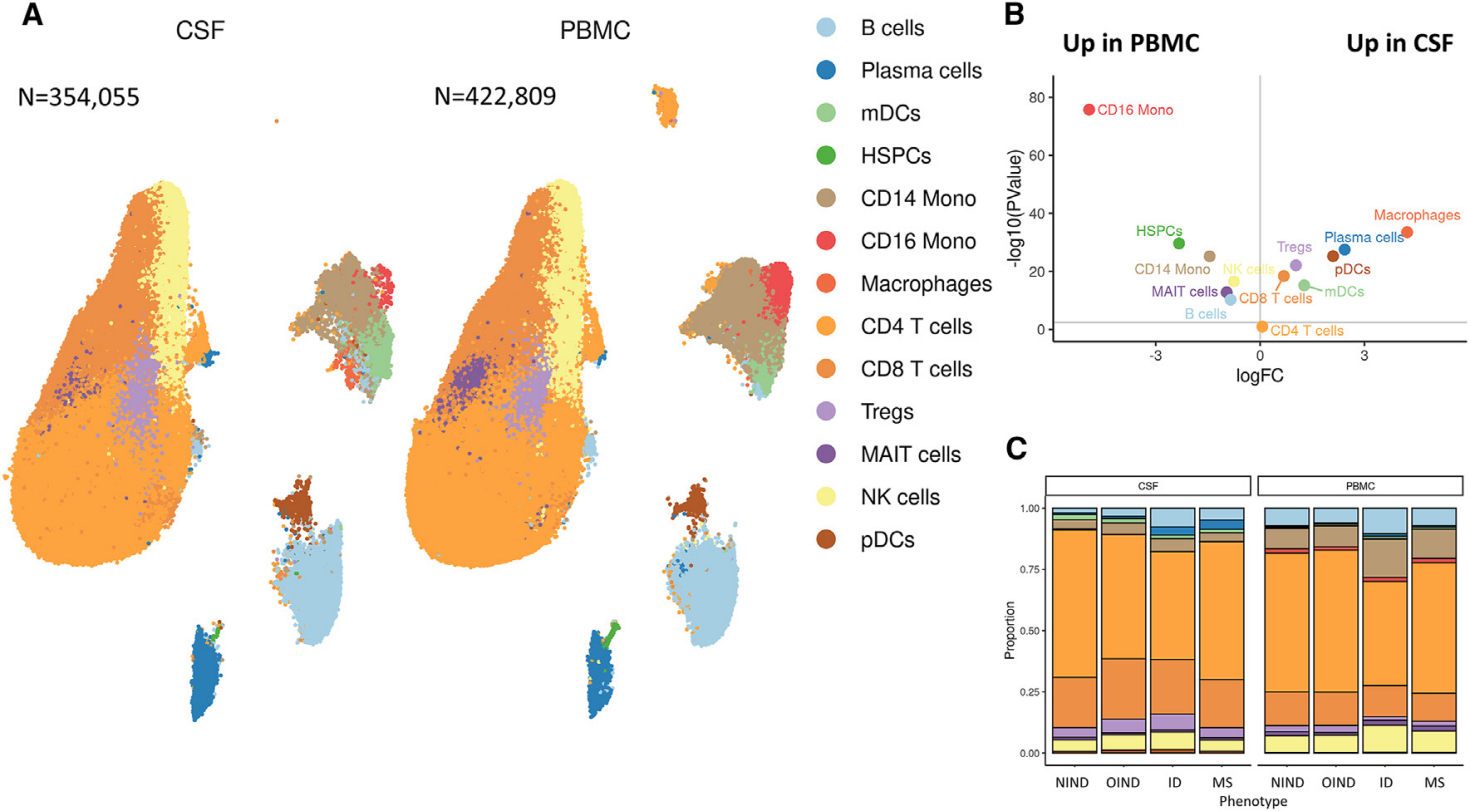
The cellular composition of the CSF is notably different from that seen in PBMC (A) Uniform manifold approximation and projection (UMAP) plot displaying individual cells in the single-cell dataset colored according to cell type. (B) Volcano plot showing differential abundance comparing the cell type proportions in CSF vs. PBMC (pooled across all disease cohorts). The x axis shows the log-fold change (logFC) in cell type proportion, with positive values indicating a higher proportion in CSF compared with PBMC. The y axis shows the −log10 of the *p* value, with values above the horizontal gray line achieving statistical significance at a Bonferroni-adjusted *p* value threshold (alpha 5%). (C) Bar plot showing cell type proportions in CSF and PBMC in each cohort separately. Abbreviations: NIND, non-inflammatory neurological diseases; OIND, other inflammatory neurological diseases; ID, infectious neurological diseases; MS, multiple sclerosis; CSF, cerebrospinal fluid; PBMC, peripheral blood mononuclear cell.

We generated single-cell RNA sequencing data from 354,055 CSF cells (see Figure 1A and Table 1)—collected from 123 largely untreated people with MS (203,220 cells), 19 patients with other inflammatory neurological disorders (OINDs; 30,796 cells), 23 patients with infectious neurological disorders (IDs; 83,339 cells), and 36 patients with non-inflammatory neurological disorders (NINDs; 36,700 cells). We also collected venous blood from a subset of these individuals and generated equivalent RNA sequencing data for 422,809 cells from the peripheral circulation (PBMC)—including 76 patients with MS (310,851 cells), 12 with OIND (25,112 cells), 4 with ID (30,299 cells), and 28 with NIND (56,547 cells; Figure 1A; Tables 1 and Table S1. Numbers of cells analyzed by compartment and disease status, related to Figure 1 and Table 1, Table S2. Numbers of donors according to site, compartment, and disease state, related to Figure 1 and Table 1, Table S3. Demographics of included participants and cell numbers per sample, related to Figure 1 and Table 2; Figures S1–S4).

**Table 1:** Demographic characteristics of included participants

Variable	MS	NIND	OIND	ID
Total	126	40	19	23
Age (median [IQR])	32.5 (16)	42 (25)	49 (14)	41 (19)
Gender (*n* [%])				
F	85 (67.5%)	25 (62.5%)	7 (36.8%)	9 (39.1%)
M	41 (32.5%)	15 (37.5%)	12 (63.2%)	14 (60.9%)
CSF OCBs (n [%])				
Negative	4 (3.3%)	15 (83.3%)	13 (68.4%)	4 (50%)
Positive	119 (96.7%)	3 (16.7%)	6 (31.6%)	4 (50%)

The cellular composition of CSF differed substantially from PBMC (Figures 1A and 1B; Tables 2 and Table S3. Demographics of included participants and cell numbers per sample, related to Figure 1 and Table 2, Table S4. Cluster annotations at “low” and “high” levels including CellTypist consensus annotations and cluster-defining genes, related to Figure 1 and Table 2, Table S5. Differential abundance results, related to Figure 1 and Table 2). In the absence of inflammation—i.e., in the cohort of patients with NINDs such as primary headache syndromes and idiopathic intracranial hypertension—CSF was enriched with dendritic cells (DCs), CD8^+^ T cells, monocyte-derived CSF macrophages, and regulatory T cells (T_regs_, false discovery rate [FDR] < 0.01) and depleted of B cells and monocytes compared with the peripheral blood.^7^^,^^12^^,^^17^^,^^18^ In the context of inflammation—i.e., in the MS, OIND, and ID cohorts—the CSF was notably enriched for plasma cells and plasmablasts—referred to collectively as “antibody-secreting cells” (ASCs) throughout—with this enrichment being particularly marked in the context of MS and ID (Table 2; Figure 1C; Table S3. Demographics of included participants and cell numbers per sample, related to Figure 1 and Table 2, Table S4. Cluster annotations at “low” and “high” levels including CellTypist consensus annotations and cluster-defining genes, related to Figure 1 and Table 2, Table S5. Differential abundance results, related to Figure 1 and Table 2).^2^^,^^7^^,^^12^

**Table 2:** CSF and PBMC cell type proportions in each of the four disease groups

	CSF				PBMC			
Cell type	MS	NIND	OIND	ID	MS	NIND	OIND	ID
B cells^a^	3.2% (3.7)	1.3% (1.8)	2.6% (3.4)	3.4% (4.1)	6.7% (3.5)	7.5% (2.9)	6.6% (5.6)	8.2% (7.1)
Plasma cells/ASCs^a^	1.7% (2.1)	0.2% (0.6)	0.8% (1.1)	1% (2.4)	0.4% (0.4)	0.3% (0.2)	0.2% (0.5)	0.9% (0.4)
mDCs	1.3% (1.4)	1.5% (1.3)	2.3% (2.4)	1.2% (1.9)	0.6% (0.6)	0.4% (0.4)	0.4% (0.6)	1% (0.4)
HSPCs	0% (0)	0% (0)	0% (0)	0% (0)	0.1% (0.1)	0.1% (0.1)	0.1% (0.1)	0.2% (0.1)
CD14 mono	2.7% (2.5)	4% (4.2)	4.5% (5.1)	2.5% (1.6)	10.8% (7.9)	8.1% (4.8)	7.7% (7.3)	15.4% (6.1)
CD16 mono	0% (0)	0% (0.1)	0% (0)	0% (0)	1.6% (1.5)	1.6% (1.6)	1.2% (1)	1.5% (0.4)
Macrophages	0.2% (0.5)	1.2% (1.2)	0.4% (0.5)	0.1% (0.1)	0% (0)	0% (0)	0% (0)	0% (0)
CD4^+^ T cells	58% (13.7)	59.8% (9)	52.1% (15.3)	45.6% (17.9)	52.7% (14.4)	54.5% (13.5)	58.2% (6.6)	42.9% (6.4)
CD8^+^ T cells	20.1% (8)	21.5% (7.6)	17.7% (14.5)	22.7% (9.8)	10.3% (5.3)	13.8% (7.4)	12.6% (8.2)	12.7% (1)
T_regs_	3.8% (2.8)	4.2% (2.2)	4.2% (3.8)	6% (4.7)	1.8% (1.3)	2.3% (1.2)	2.5% (1.8)	1.2% (0.4)
MAIT cells	0.7% (0.7)	0.8% (0.9)	0.8% (0.9)	0.8% (1.4)	1.5% (1.3)	1.3% (1.4)	1% (1.1)	1.5% (2)
NK cells	4.3% (2.4)	3.5% (2.1)	3.8% (3.8)	6.1% (4.4)	7.8% (6.3)	6.5% (3.4)	6.6% (3.3)	9.4% (5.5)
pDCs	0.6% (1)	0.3% (0.9)	0.8% (1)	0.7% (1)	0.2% (0.2)	0.2% (0.2)	0.1% (0.2)	0.3% (0.1)

Values represent the median % of the total PBMC/CSF cell population in each disease cohort (i.e., the percentage of the PBMC/CSF cell population was calculated per person, and then the median was taken across the cohort). The values in brackets represent the interquartile range. Only subjects with >10 total cells in the relevant compartment (PBMC or CSF) were included in these calculations.

a Indicates the two cell types—B cells and plasma cells/ASCs—which were present at higher proportions in MS CSF compared with NIND CSF at a false discovery rate of <1%.

We observed striking heterogeneity of both PBMC and CSF cell type proportions between individuals (Tables S3 and S5; Figures S5 and S6). For example, within the MS cohort, the proportion of ASCs in CSF varied from 0.05% to 25.1% (median 1.7%, interquartile range [IQR] 2.1%). We considered whether this heterogeneity was related to biological differences in disease characteristics or merely reflective of inherent variability between individuals. As expected, the proportion of ASCs was higher in MS patients with CSF oligoclonal bands (*n* = 119) than in those without bands (*n* = 4) (FDR < 0.1, ∼6.2×-fold increase), and there were no statistically significant changes in other cell type proportions. Comparison of patients with primary progressive MS (PPMS, *n* = 8) with relapse-onset MS (RMS, *n* = 113) did not show any suggestive (FDR < 0.1) differences in CSF cell type proportions, arguing for a broadly similar CSF cellular landscape in these clinically defined disease categories. Analysis of ligand-receptor co-expression suggested roles for *CXCR3* and *CXCR4* as plausible mediators of increased B cell and ASC entry into the CSF (Figure S7). By contrast, no significant differences were observed between MS and any of the ID, OIND, or NIND in the peripheral blood compartment (Figure 1C). MS CSF was compositionally similar (i.e., no differences at FDR < 1%) to OIND CSF but contained lower relative proportions of natural killer (NK) cells and T_regs_ compared with ID CSF (Table S5). We did not find evidence for the presence of MS-specific cell types.

### Gene expression in the intrathecal compartment reveals a central role for cholesterol homeostasis

**Figure 2:**
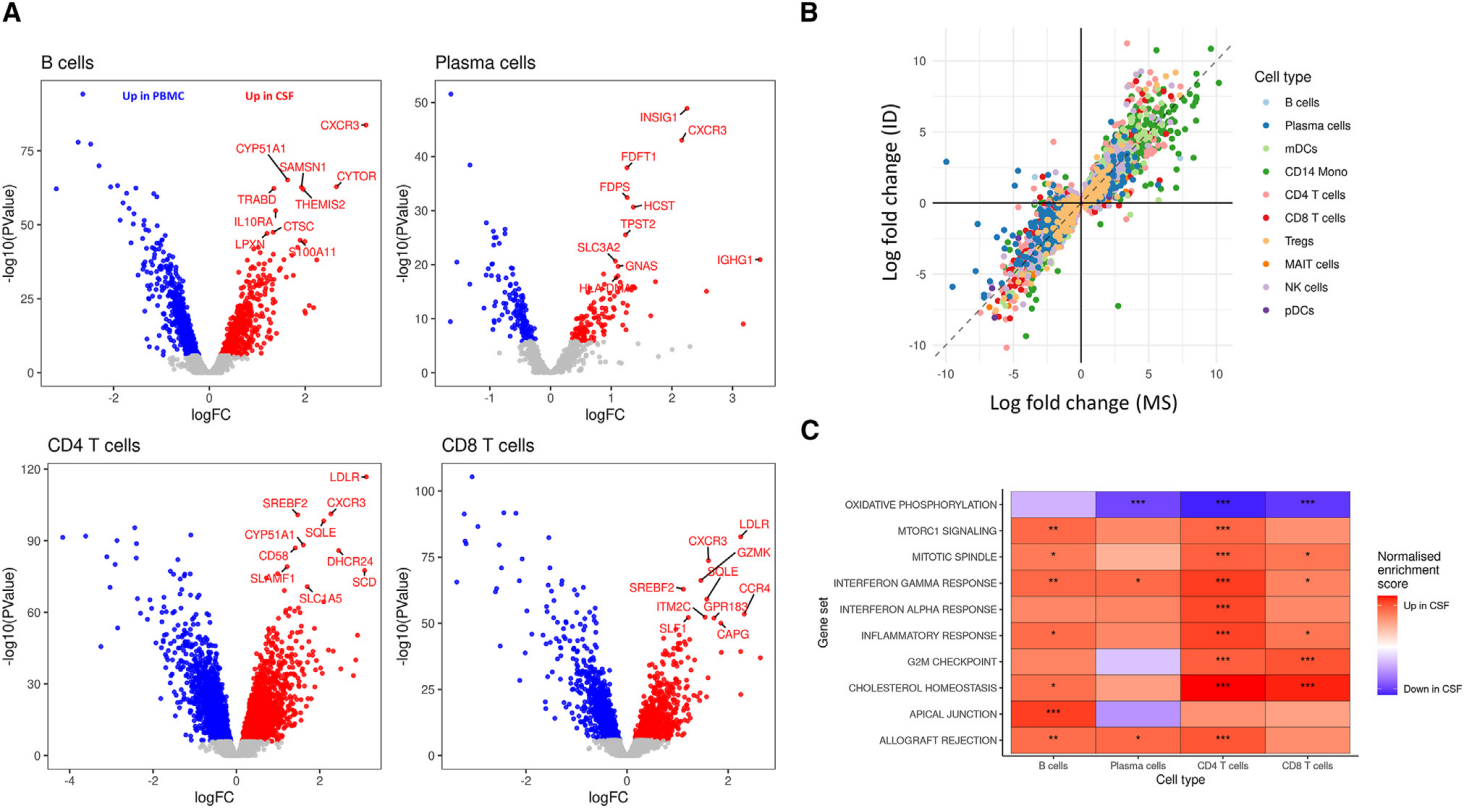
Transcriptional profiling of CSF leukocytes reveals tissue-specific gene expression (A) Volcano plot displaying results of differential expression testing comparing gene expression in CSF and PBMC for four selected cell types of interest (pooling data across disease cohorts). Each dot represents a gene tested, the y axis shows the −log10(*p* value), and the x axis shows the log2-fold change in transcript abundance. Genes colored in red are upregulated in CSF while genes colored in blue are downregulated. Tests with a Bonferroni-corrected *p* value greater than 0.01 are shown in gray. Note that the y axes are on different scales for clarity. (B) Scatterplot comparing differential expression results in MS and ID CSF. Each dot is a gene. The x axis shows the log-fold change from MS CSF to MS PBMC (i.e., positive values indicate upregulation in MS CSF compared with PBMC), and the y axis depicts the log-fold change in ID CSF versus ID PBMC. Dots are colored according to cell type in which they were tested. Only genes achieving statistical significance in MS are shown. The dotted line represents the null hypothesis that the change in gene expression in CSF is identical between MS and ID. (C) Gene set enrichment analysis (GSEA) results comparing the expression of genes involved in Hallmark canonical pathways in CSF vs. PBMC across multiple cell types. The tiles are colored by the direction of their normalized enrichment score (NES), with red (positive) indicating upregulation in CSF and blue (negative) indicating downregulation. ∗, FDR < 0.05; ∗∗, FDR < 0.005; ∗∗∗, FDR < 0.0005. Again, these results reflect the pooled analysis, i.e., comparing all CSF samples with all PBMC samples. Cohort-specific results are presented in Table S6.

Differential gene expression analysis showed that, in comparison to PBMCs, CSF immune cells upregulated markers of tissue residence, cytotoxicity, and antigen presentation, consistent with previous reports.^11^^,^^12^ This upregulation was seen in multiple cell subtypes (Figure 2A; Table S6; Figure S8)^6^^,^^8^^,^^11^^,^^12^ and was largely independent of disease context (i.e., there was strong concordance between MS, ID, and OIND cohorts; Figure 2B). Gene set enrichment analysis (GSEA) showed upregulation of genes involved in cholesterol homeostasis in the majority of cell types, and of MTORC1 signaling in B cells and ASCs (Figure 2C). Weighted pathway analysis implicated the sterol regulatory element binding protein transcription factors *SREBF1* and *SREBF2* as the likely drivers of these changes. These transcription factors (*SREBF1* and *SREBF2)* regulate intracellular fatty acid and cholesterol synthesis in lymphocytes and thereby determine the supply of substrate necessary for proliferation and clonal expansion.^19^^,^^20^^,^^21^

To systematically search for MS-associated gene expression, we performed differential expression testing comparing MS CSF with OIND and ID CSF. Compared with ID CSF, MS CSF displayed upregulation of a small number of genes, including *CCL22* in B cells and ASCs, *CD99* in myeloid DCs (mDCs), *CRIP2* in CD4^+^ T cells, and *LEKR1* in CD14^+^ monocytes (Table S6). There were very few differences between MS and OIND CSF, suggesting that the CSF alterations observed in MS are not disease specific but are very similar to inflammatory changes observed in other inflammatory central nervous system (CNS) diseases involving humoral immune responses.

### Transcriptional features of B cell clonal expansion across diseases

**Figure 3:**
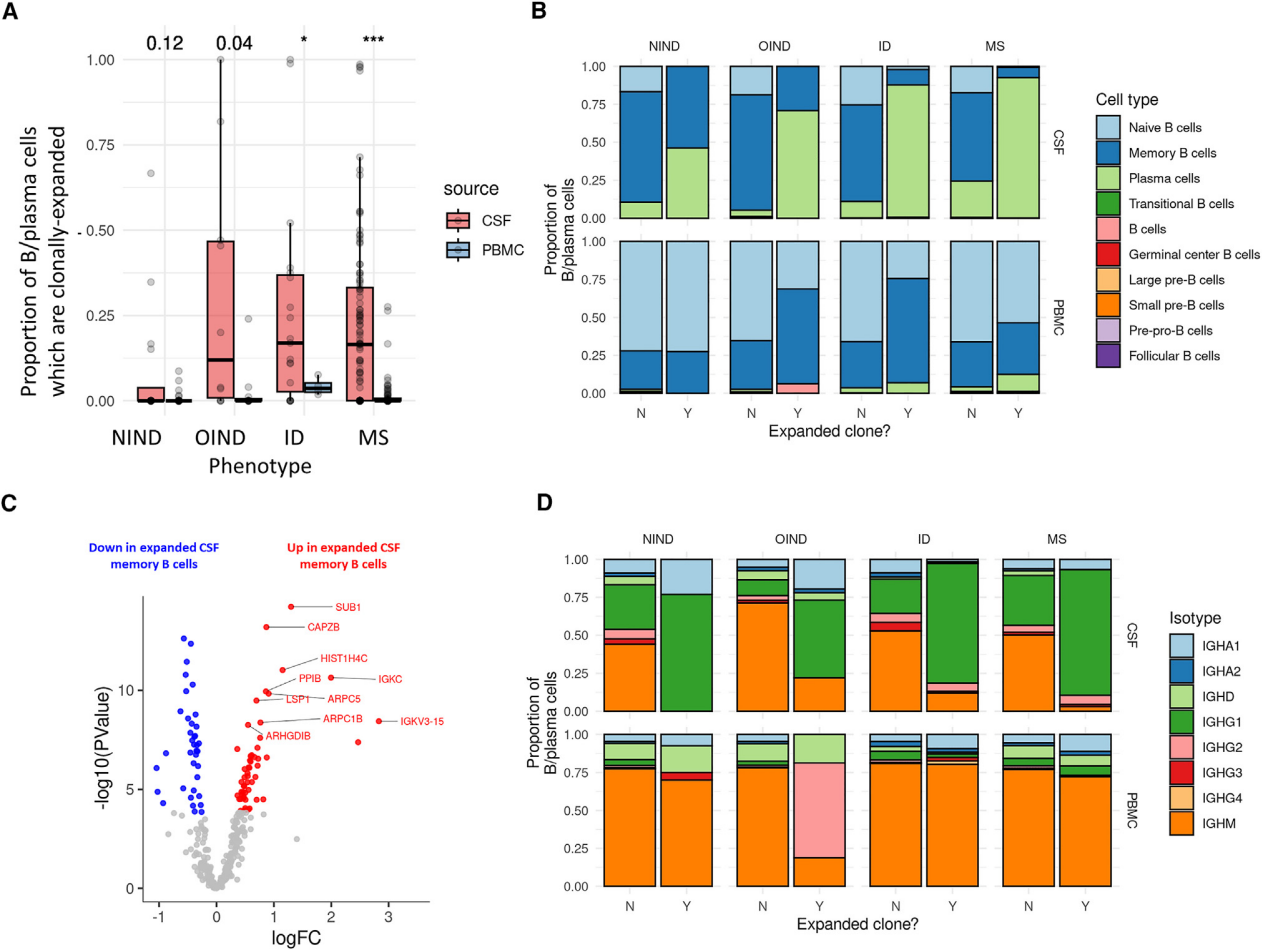
The nature of clonally expanded B cells (A) Barplots showing the proportion of B cells and ASCs in each disease cohort and each of CSF/PBMC which were part of an identified expanded clonal group. The numbers at the top of the plot indicate the *p* values for the comparison of clonal % between CSF and PBMC in each disease cohort. ∗, *p* < 0.01; ∗∗∗, *p* < 0.0001. (B) Barplot showing the overall cellular composition of the expanded vs. non-expanded B cell pool, highlighting the observation that the expanded pool is primarily composed of ASCs. (C) Volcano plot showing differential expression results contrasting clonally expanded vs. non-expanded CSF memory B cells in a pooled analysis of all disease cohorts. (D) As per (B), but showing the isotypes expressed by expanded vs. non-expanded cells, showing the marked shift toward IgG isotypes, particularly IgG1 among expanded cells.

Focusing on B-lineage cells, we found that those present in the CSF were more frequently class-switched, antigen-experienced cells (largely memory cells and ASCs) and showed greater levels of somatic hypermutation than peripheral B cells, in keeping with the idea that most of these cells have experienced antigenic stimulation within the intrathecal compartment (Figure 3).^12^ CSF B cells/ASCs were enriched for the immunoglobulin G1 (IgG1) isotype in the context of inflammatory and infectious CNS disorders compared with the peripheral blood (Figure 3). In keeping with previous reports, we observed preferential usage of the *IGHV4* immunoglobulin heavy-chain gene segment families in MS CSF compared with peripheral blood, and a bias toward immunoglobulin light-chain kappa gene segments (Table S11; Figure S8).^2^^,^^14^^,^^16^ Neither the *IGHV4* bias nor the *IGKV* bias was observed in either the OIND or ID cohorts (Table S11), and so these findings may represent an MS-specific predilection of the intrathecal B cell repertoire. To avoid statistical bias due to selective usage of specific *IGHV* or *IGKV* segments by highly expanded clones, each clone was sampled only once for these analyses. At the more granular level of individual gene segments, we had less statistical power (due to both the smaller number of cells with each gene segment and the greater burden of multiple testing). However we found suggestive evidence that the *IGHV4* bias was driven by overexpression of *IGHV4-31* (log2-fold change [logFC] 0.96, *p* = 0.01, FDR = 0.12) and that the *IGKV* bias was driven by several segments (including *IGKV6-21*, *IGKV1-27*, *IGKV2-28*, and *IGKV1-33* all with FDR < 0.1), of which the strongest effect was observed for *IGKV6-21* (logFC 1.4, *p* = 2.1 × 10^−6^, FDR = 3.2 × 10^−4^).

Based on canonical receptor sequence homology, we identified 3,126 clonally expanded B cells (3,126/22,964, 13.6%) belonging to 602 sets of clonally related B cells (449 were found in the CSF alone, 151 in the peripheral blood alone, and 2 spanned both compartments). The proportion of clonotypic B cells was much higher in the CSF of patients with OIND, MS, and ID compared to peripheral blood. While few subjects in the NIND (4/28, 14.3%) cohort had detectable CSF B cell clones, these were commonplace in the OIND (7/15, 46.7%), ID (14/23, 60.9%), and MS cohorts (62/116, 53.4%; note that denominators reflect the number of patients with any detectable B cells in their CSF and so are slightly smaller than the total number of CSF samples), reiterating the concept that CSF B cell clonal expansion is a general feature of intrathecal inflammation, and not specific to MS (Figure 3A). Although most of the clonal groups we observed consisted of just two clonally related cells (357/602, 59.3%), some clones were much larger with over 30 observed cells. These highly expanded clones were only observed in the MS and ID cohorts. However, given the strong correlation between number of cells sampled and clonal size, this may merely reflect a sampling bias.

In the CSF most clonally expanded B-lineage cells (75.2%) were IgG1+ ASCs that showed evidence of somatic hypermutation. Clonally expanded cells showed a subtle enrichment in the usage of *IGHV4* heavy-chain segments and *IGHK* light chains (Figures 3B, 3C, and S9). When we categorized the patients by disease subgroup, we found that these effects were driven by the MS cohort: clonally expanded cells in the MS patients showed biases toward usage of the *IGHV4-39* and *IGHV4-59* gene segments, whereas this effect was not observed in the ID, OIND, or NIND patients. We observed a similar phenomenon for the light-chain segments: in the MS CSF, clonally expanded cells showed biased usage of *IGKV1-9*, *1-13*, *1-17*, and *3-15*, none of which were upregulated in clonal cells from the other disease cohorts (Figure S10).

We next performed differential expression analysis comparing expanded vs. non-expanded cells within each cell type in each compartment (Table S12). We performed these analyses in the pooled cohort, combining MS, ID, OIND, and NIND samples. We detected two genes which were upregulated in CSF expanded ASCs—*AL138963.4*, a long noncoding RNA of unknown function, and *HIST1H1D*, which encodes a histone protein. This histone protein is a target for driver mutations in myeloma and B cell lymphomas^22^ and so is a plausible driver of ASC clonal expansion. Within the CSF memory B cell cluster, we identified 89 genes which were differentially expressed in clones (Figure 3D; Table S7, *P*_Bonferroni_<0.05, 52 upregulated, 37 downregulated). The most upregulated transcript was *SUB1* (log-fold change 1.3, *p* = 2.1 × 10^−12^), the gene product of which—positive coactivator 4 (PC4)—promotes ASC differentiation and maturation.^23^ Other upregulated genes in clones included genes involved in antigen presentation via class II major histocompatibility complex molecules (*HLA-DRA*, *HLA-DPA1*, *B2M*, *IFI30*), cytoskeletal architecture (*TMSB4X*, *ARPC1B*, *ARPC5*, *CAPZB*, *LSP1*, *HCLS1*), and guanosine triphosphate-binding proteins (*ARHGDIB*, *RAC2*). Weighted pathway analysis suggested increased activity of *RFX5* targets in clonally expanded memory cells (*p* = 0.01), largely driven by upregulation of *B2M*, *CD74*, and class II histocompatibility leukocyte antigen (HLA) genes (*HLA-DPA1*, *HLA-DRA*, *HLA-DRB1*, *HLA-DPB1*, and *HLA-DQA1*). To determine whether these changes were common across diseases, we repeated the analysis stratified by disease. We found that the effect was largely attributable to the MS and ID groups, with consistent effects of the top clone-defining genes (e.g., *SUB1*, *CAPZB*, and *ARPC5*) in both MS and ID expanded CSF memory cells (Tables S7 and S12). These findings suggest a common transcriptional signature associated with B cell clonal expansion across different disease contexts. *SUB1* expression was associated with increased expression of B cell maturation markers (Figure S11) and showed a gradient of expression from naive B cells (lowest) to plasma cells (highest), indicating that the clone-defining genes we identify likely reflect active B cell differentiation into ASCs.

### Characteristics of the CSF T cell repertoire in neuroinflammation

**Figure 4:**
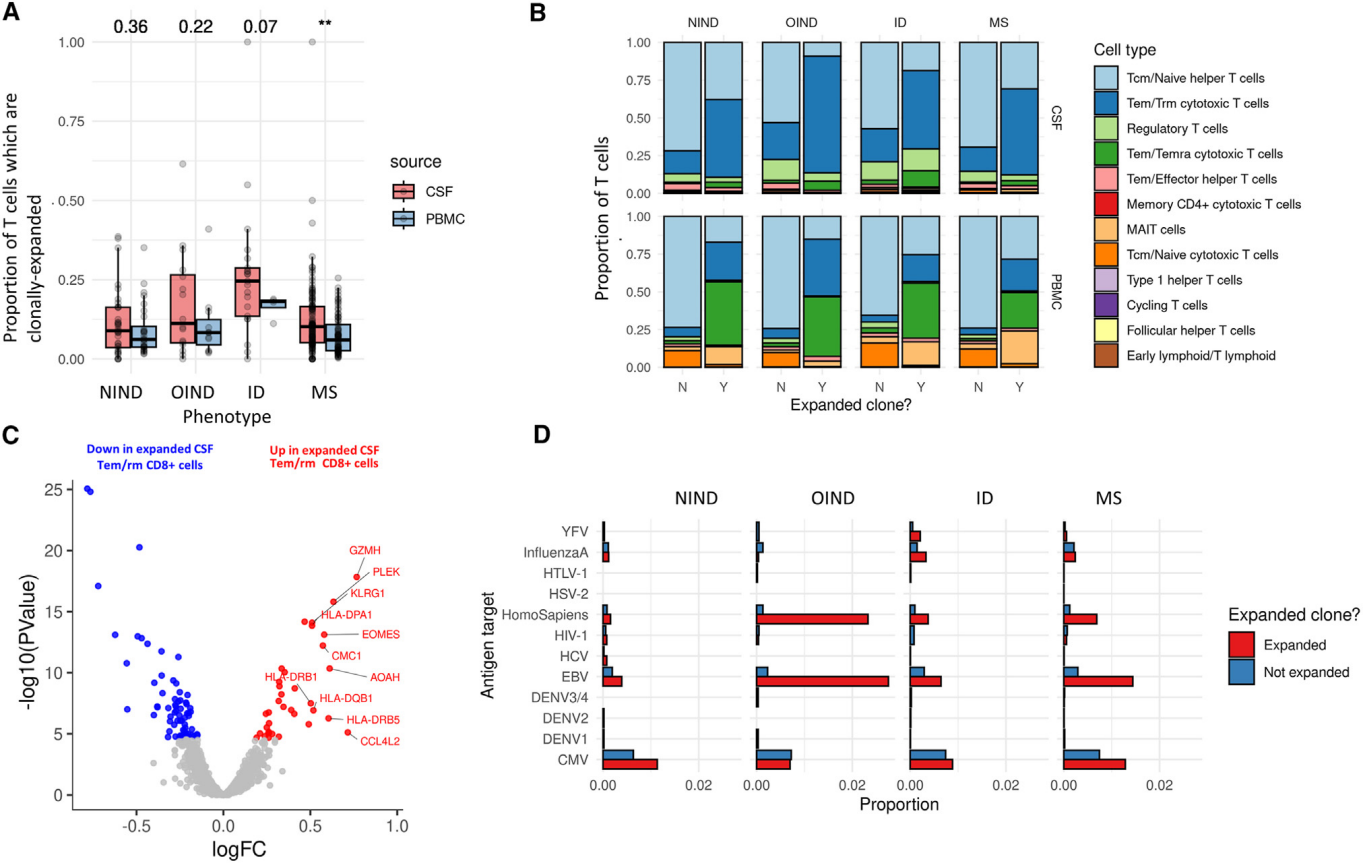
The TCR repertoire in neuroinflammation (A) Boxplots showing the proportion of clonally expanded T cells in the CSF and PBMC of each disease group. Numbers at the top of the plot show the *p* values for the comparison of CSF vs. PBMC. ∗, *p* < 0.01; ∗∗, *p* < 0.001; ∗∗∗, *p* < 0.0001. (B) Barplots showing the cellular composition of the clonally expanded vs. non-expanded T cell pools in CSF and PBMC. (C) DE volcano plot contrasting gene expression in MS CSF T resident memory T (Trm) cells vs. non-expanded cells, highlighting upregulation of cytotoxicity markers and HLA molecules. (D) Boxplot showing the proportion of T cells within the expanded and non-expanded subsets with a TCR beta chain CDR3 predicted to bind various epitopes in each cohort divided by clonal status (data are shown for CSF TCRs only). Clonally expanded TCRs were more likely to recognize Epstein-Barr virus (EBV) antigens in both MS and controls.

The T cell composition of CSF was distinguished from PBMC by a statistically significant shift (FDR <1%) toward memory, effector, and resident phenotypes (T_EM/RM_ cytotoxic T cells, type 1 helper T cells, T_regs_, follicular helper T cells, memory CD4^+^ cytotoxic T cells, and T_EM/Effector_ CD4^+^ T cells), and a relative depletion of naive T cells (both CD4^+^ and CD8^+^), mucosal-associated invariant (MAIT) T cells, and T_EM_/T_EMRA_ cytotoxic T cells (Table S8). We explored the relationship between changes in CSF T cell composition and phenotype and found that most of these alterations were features of the CSF T cell pool in general and were not specific to MS. The increase in T follicular helper cells was observed in all three inflammatory cohorts (MS, OIND, and ID), but not in the non-inflammatory controls, suggesting that the presence of these cells in CSF may be a feature of neuroinflammatory CNS disorders (Figure 4B).

We identified 37,673 clonally expanded T cells in the dataset derived from 11,541 expanded clonotypes. Unlike in the B cell compartment, we observed extensive clonal sharing across the blood-CSF barrier, with 986 clones observed in both CSF and PBMC. Again in contrast to the B cell pool, the degree of clonality of the CSF T cell compartment did not differ significantly between patients with MS and NIND and in fact was lower in MS than in the ID cohort (Figure 4A, *p* = 0.01). Clonally expanded T cells were dominated by effector memory CD8^+^ cytotoxic subsets (Figure 4B) in both blood and CSF. In the peripheral blood, the clonal T cell population was enriched for T_EM/RM_ (effector memory/resident memory) CD8^+^ cytotoxic cells, T_EM_/T_EMRA_ (effector memory/effector memory re-expressing CD45RA) CD8^+^ cytotoxic cells, and MAIT cells compared with the non-expanded pool. In contrast the majority of clonally expanded CSF T cells were of a T_EM/RM_ phenotype (>50%), with a lesser degree of enrichment for T_EM_/T_EMRA_ cells, and no enrichment of MAIT cells (Figure 4B).

As with the B cell compartment, clonally expanded cells showed preferential usage of specific T cell receptor beta variable (*TRBV*) gene segments (*TRBV6-4*, *TRBV7-8*, and *TRBV27*) and T cell receptor alpha variable (*TRAV*) gene segments (*TRAV1-2*, *TRAV14*, *TRAV19*, and *TRAV38-2*). These findings were not solely driven by MAIT cells (which classically express *TRAV1-2* with either *TRBV6* or *TRBV20* genes^24^), as exclusion of these cells yielded similar results. Differential expression analysis comparing expanded and non-expanded T cells revealed upregulation of cytotoxicity markers (*GZMA*, *GZMK*, *GZMH, GZMM*, *PRF1, CST7*, *NKG7*, *CD8A)*, chemokines and/or chemokine receptors (*CCL5*, *CXCR3*), and also markers of senescence/exhaustion (*KLRG1*) across multiple cellular subtypes (Figure 4C). GSEA identified enrichment of interferon γ signaling and complement pathways in expanded clones across multiple cell types. These clone-defining genes were similar across cohorts, arguing for a generic transcriptional program associated with clonal expansion rather than an MS-specific phenomenon.

We found 736 T cell clonotypes that were seen in more than one individual. Many of these clones were MS specific, i.e., only observed in the MS cohort; however, we also observed many clones specific to the ID cohort. Considering CDR3 similarity alone to predict epitope binding, we found that MS CSF was enriched with Epstein-Barr virus (EBV)-specific TCR CDR3 sequences compared with NIND CSF (FDR < 0.01) but contained a lower proportion of EBV-specific sequences than the OIND cohort. We obtained similar results using a more stringent definition of EBV-specific, stipulating that both the CDR3 amino acid sequence and the *TRBV* gene usage must match the reference dataset (Table S13). Importantly, the high frequency of T cells predicted to bind EBV epitopes was neither specific to the MS cohort nor specific to EBV; we observed similarly high levels of predicted viral-reactive T cells for cytomegalovirus (CMV) (Table S13), suggesting that this observation does not reflect a pathogen-specific response. Clonally expanded T cells were enriched for TRB-CDR3s predicted to bind to a variety of epitopes—including EBV and CMV epitopes—in both the MS and the control cohorts (Figure 4D).^25^

### CSF-specific genetic control of gene expression

The distinct transcriptional profile of CSF leukocytes offers an opportunity to explore the genetic control of gene expression in the intrathecal compartment. Genes which are highly expressed in CSF, but not in blood, are under-represented in existing expression quantitative trait locus (eQTL) datasets.^26^^,^^27^^,^^28^ We therefore performed *cis*-eQTL mapping in CSF and peripheral CD4^+^ T cells, CD8^+^ T cells, and B cells, focusing on 2,499 prioritized genes which were upregulated in CSF, have been implicated in MS pathogenesis through susceptibility genome-wide association studies (GWASs),^29^ or have been identified as regulators of T cell migration to the CSF.^30^ Most of the eQTLs we observed have been reported before^26^^,^^27^^,^^28^ (see Figure S12). eQTL effect sizes were correlated between CSF and PBMC (*r*^*2*^ = 0.72) and across different cell types (*r*^*2*^ = 0.45), supporting the concept that many eQTLs act in a similar manner across different compartments and cell types.

We considered whether MS susceptibility alleles could exert CSF-specific effects on gene expression. We found several genes with strong evidence for colocalization (*ORMDL3*, *ANKRD55*, *FCRL3*, *AHI1*, *EAF2*, *GDPD5*, and *ZC2HC1A*; see Table S10), i.e., instances where the MS risk allele also alters expression of these transcripts in the CSF. All of these genes were differentially expressed between CSF and peripheral blood cells in single or multiple cell types in our data (FDR < 5%, see Table S6). Several of these effects appear to be cell type specific. For instance, rs4676755 (in perfect linkage disequilibrium with the MS risk variant rs2331964) exerts an apparently B cell-specific effect on *EAF2* expression, with each copy of the risk allele decreasing *EAF2* expression. We also report that the MS risk SNP rs1466526 is likely to act as an eQTL for *ZC2HC1A* in CSF B cells and CD4^+^ T cells with the risk allele (rs1466526-C) decreasing *ZC2HC1A* expression. Although this eQTL was reported in eQTLGen and in a recent single-cell sequencing study of PBMC,^26^^,^^28^ colocalization with an MS risk signal has not previously been shown. In our study, *ZC2HC1A* is expressed almost exclusively in CSF cells (expressed in less than 2% of all PBMCs). *ZC2HC1A* encodes a zinc-finger protein of which decreased brain levels, but not plasma levels, are associated with MS susceptibility.^31^ These observations highlight the value of studying CSF to understand eQTL effects in disease-relevant tissues.

**Figure 5:**
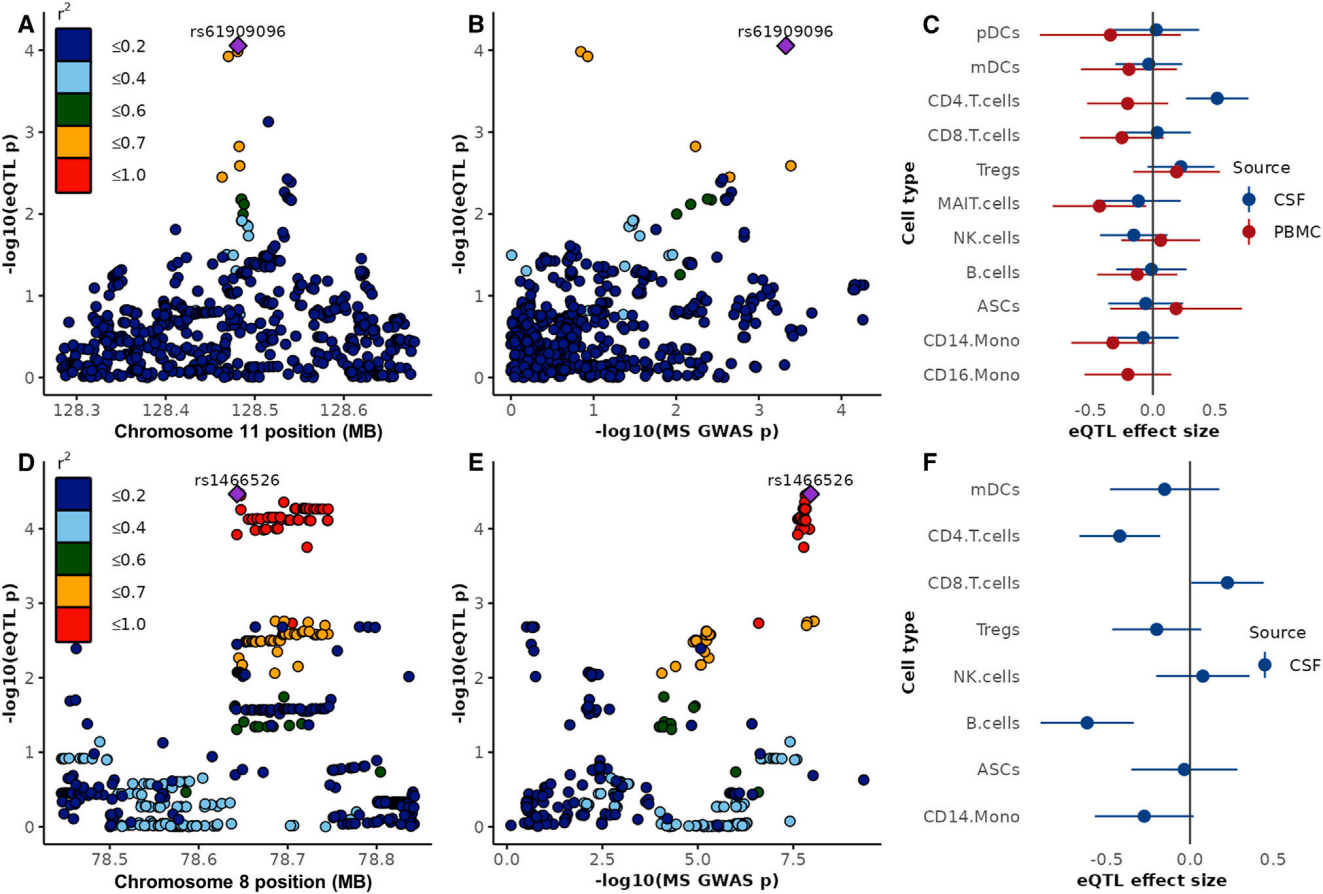
CSF cell eQTL analysis (A) Regional association plot for a previously unknown eQTL on chromosome 11 for *ETS1* expression CSF CD4^+^ T cells. Each dot represents one tested single nucleotide polymorphism (SNP), colored by the degree of linkage disequilibrium (LD, *r*^*2*^) to the lead SNP. (B) Correlation of eQTL *p* values and *p* values for MS risk (IMSGC 2019 susceptibility GWAS) for the same locus on chromosome 11. (C) Forest plot showing the eQTL effect estimates +95% confidence intervals of the lead SNP rs61909096 on *ETS1* expression in different cell types and compartments, suggesting a specific effect for CSF CD4^+^ T cells. (D) Regional association plot for the locus around a known eQTL on chromosome 8 associated with *ZC2HC1A* expression in CSF B cells that colocalizes with an MS risk signal. (E) Correlation of eQTL *p* values and *p* values for MS risk (IMSGC 2019 susceptibility GWAS) for the same locus on chromosome 8. (F) Forest plot showing the eQTL effect estimates +95% confidence intervals of rs1466526 on *ZC2HC1A* expression in 8 cell types.

We identified three apparently CSF-specific eQTLs (see Table S9; Figure S13). These eQTLs have not been previously reported^26^^,^^28^^,^^32^^,^^33^ and therefore require external replication. They include an eQTL for *ETS1* in CSF CD4^+^ T cells (*p* = 8.8 × 10^−5^), a transcription factor recently identified as one of five essential brakes on T cell migration to the CNS in MS^30^ (Figure 5). Interestingly, this eQTL has not been reported before at a significant or even suggestive level, but a nominal association of the lead variant rs61909096 with *ETS1* expression in brain (*p* = 0.02) and the pituitary gland (*p* = 0.04)^27^ as well as peripheral CD4^+^ T cells (*p* = 0.03)^34^ has been reported.^33^ While we did not observe colocalization of this eQTL with any known MS risk signal, the eQTL lead SNP rs61909096 is nominally associated with MS risk (*p* = 4.7 × 10^−4^).^29^ The MS risk allele (rs61909096-G) reduces the expression of *ETS1* in CSF T cells, which may potentiate T cell entry into the CNS.^29^^,^^30^

## Discussion

In this study we describe the immune landscape of CSF in a range of neuroinflammatory disorders and non-inflammatory controls at single-cell resolution. Using a combination of genetics, transcriptomics, and lymphocyte receptor repertoire sequencing, we provide a detailed description of the intrathecal immune response in the context of neuroinflammation.

In keeping with previous studies, we confirm that the CSF in MS is characterized by an increased representation of ASCs and memory B cells compared with healthy controls and peripheral blood.^6^^,^^8^^,^^12^^,^^35^^,^^36^ In contrast to a previous single-cell sequencing study, we were unable to identify any cell subsets which were specific to MS.^13^ These profound alterations in the cellular composition of CSF were not mirrored in peripheral blood, where we found no substantial changes in cell composition in the MS cohort compared with the non-inflammatory neurological disease cohort. We found that increased levels of ASC and B cell infiltration into the CSF were also evident in patients with other CNS inflammatory disorders, and in infectious disorders, suggesting that these changes are indicators of neuroinflammation, but not specific to MS.

We observed upregulation of markers of tissue residence, antigen presentation, cytotoxicity, and proliferation across cell types and across cohorts. We also observed upregulation of genes involved in cholesterol synthesis in CSF leukocytes, as has been reported previously,^11^^,^^12^ changes that are most likely driven by the transcription factors *SREBF1* and *SREBF2.* It has been postulated that cholesterol biosynthesis couples energy sensing with lymphocyte activation, provides obligate materials for proliferation, and may provide a metabolic checkpoint for T cells prior to antigen-driven clonal expansion.^21^^,^^37^ The majority of genes upregulated in CSF lymphocytes were common across the diseases studied, suggesting that these are markers of tissue-specific transcriptional programmes rather than being disease specific. We also discovered a small number of genes which appear to be upregulated in MS CSF compared with ID CSF, suggesting possible roles in disease pathogenesis. MS CSF B cells and ASCs upregulate *CCL22* compared with B cells from patients with neurological infectious diseases. *CCL22*-expressing B cells were extremely rare in the dataset (*n* = 161), and were also observed in the control cohorts, but interestingly were only present in CSF. The majority of these cells were annotated as memory B cells or ASCs. *CCL22* encodes the chemokine CCL22, which has been recently shown to mediate formation of germinal centers via interaction with CCR4 on follicular helper T cells, and to promote proliferation of high-affinity B cells.^38^ Other genes upregulated in MS CSF compared with either OIND or ID controls included *CD99*—a mediator of DC migration from tissues into lymph nodes^39^—in mDCs, *CRIP2*—an inhibitor of nuclear factor κB signaling^40^—in CD4^+^ T cells, and *LEKR1*—an MS susceptibility locus^41^—in CD14^+^ monocytes.

We provide an overview of the B cell receptor (BCR) and T cell receptor (TCR) repertoire in CSF and describe the characteristics of clonally expanded lymphocyte populations. The CSF B cell pool is dominated by antigen-experienced cells, many of which have undergone clonal expansion. While there was considerable inter-individual heterogeneity in the degree of clonal expansion, the CSF of patients with MS and neuro-infectious disorders was substantially more clonal than non-inflammatory controls. Clonally expanded B cells were largely of an IgG1+ ASC phenotype, with IgA-expressing and IgM-expressing ASCs rare in the CSF. We confirmed previous reports of a skew in the BCR repertoire toward specific heavy- and light-chain families in the CSF of MS patients, which was mainly driven by IgG1+ ASCs.

We discover a gene signature associated with clonally expanded memory B cells. The top clone-defining gene, *SUB1*, encodes PC4, a transcriptional master regulator with roles in regulation of DNA repair and chromatin remodeling. PC4 expression is positively regulated by *IRF4* and forms a complex with IRF4 and IKAROS—a target of lenalidomide-directed degradation in multiple myeloma—which promotes B cell differentiation into ASCs.^23^ *TMSB4X* encodes an actin-stabilizing protein which is downregulated in CSF-resident dural B cells,^42^ associated with an early immature B cell phenotype,^43^ and is a target of somatic mutations in B cell lymphoma.^44^ We observed upregulation of genes involved in the ARP2/3 complex (*APRC1B* and *ARPC5*), a highly conserved protein complex which stabilizes branching actin networks and underpins several functions in immune cells, including migration, phagocytosis, and coupling BCR/TCR activation to intracellular signaling.^45^ Deficient ARP2/3 function due to genetic deletion of *ARPC1B* causes a Wiskott-Aldrich syndrome characterized by a lower threshold for BCR activation, autoimmunity, and an expansion of the transitional B cell compartment.^45^ We hypothesize that overexpression of *ARPC1B* in clonal B cells may represent an appropriate homeostatic response to limit clonal expansion by dampening BCR signaling. *CAPZB*—another transcript upregulated in clones—is also involved in cytoskeletal stabilization. Importantly, these features of clonally expanded memory cells were common to both the MS and ID cohorts, suggesting that this is a disease-agnostic transcriptional program common to neuroinflammatory disorders, and not specific to MS. Taken together, these findings underscore the pivotal importance of cytoskeletal organization in clonal B cell proliferation, show the close similarity between clonal B cells in inflammatory and lymphoproliferative disorders, and suggest possible avenues for therapeutic targeting of these cells, for instance, with the myeloma drug lenalidomide,^46^ which has shown promise in rodent models.

We describe the features of the CSF TCR repertoire in inflammatory and non-inflammatory neurological disorders. Consistent with previous reports, we find that the CSF T cell pool is polarized toward memory and effector T cell subsets in health and disease. We identify a large number of clonally expanded T cells which are present in similar proportions in inflammatory diseases and non-inflammatory controls, with higher levels observed in neurological infections and a tendency for greater clonality in the CSF T cell pool than in the periphery.^11^ We found that neuroinflammatory CSF is enriched with T_regs_ and follicular helper T cells compared with non-inflammatory CSF; these findings were indicative of CSF inflammation rather than being MS specific. Clonally expanded cells in the CSF were predominantly of a CD8^+^ tissue-resident effector memory phenotype, in contrast to the peripheral blood where many clonally expanded cells were MAIT cells or T_EM_/T_EMRA_ cells. Clonally expanded T cells show a bias toward specific *TRAV* and *TRBV* genes and overexpress genes involved in cytotoxicity, again consistent with previous findings.^11^ Clonally expanded cells were enriched for EBV-specific and CMV-specific CDR3 sequences in both MS cases and controls, mirroring recent findings in Alzheimer’s disease.^25^ Although this may reflect a biological association between TCR specificity and clonality (i.e., it is plausible that these common viruses are a common cause of TCR activation and clonal expansion), these findings are also likely to represent the limited and biased nature of public TCR databases, which are dominated by CMV and EBV epitopes.

Finally, given that many genetic associations underlying complex traits—including MS—are thought to exert their effects by altering gene expression rather than gene function,^47^^,^^48^^,^^49^ we undertook a search for CSF eQTLs. Although there are now several biobank-scale eQTL datasets examining peripheral blood from healthy controls,^26^^,^^28^ our dataset adds value by studying CSF eQTLs in various disease contexts. We report a CSF-specific eQTL for *ETS1* in CD4^+^ T cells, a gene which has very recently been identified as one of five essential brakes for T cell migration to the CNS.^30^ We also show that the MS risk SNP rs2331964, a known eQTL for *EAF2* in B cells, also exerts this eQTL effect in CSF B cells. *EAF2* is a pro-apoptotic transcription factor which prevents excessive B cell proliferation following the germinal center reaction.^50^ This observation suggests an elegant mechanism whereby the MS risk allele decreases expression of a natural homeostatic “brake” in B cell proliferation and may therefore promote excessive proliferation of B cells into ASCs. Finally, we report colocalization between a previously reported eQTL for *ZC2HC1A* in CSF CD4^+^ T cells and MS risk. *ZC2HC1A* is predominantly expressed in CSF cells in our study and encodes a zinc-finger protein that has been associated with MS susceptibility.^31^ Our findings therefore demonstrate the value of studying eQTLs in different tissue and cell type contexts, particularly for interrogating genes which are expressed at low levels in PBMC datasets.

### Limitations of the study

There are some important limitations to this study. Despite the size of the dataset, many of our analyses—particularly those concerned with clonality and the eQTL analysis—suffer from limited statistical power due to sample size (see supplementary note on power calculations for clonal detection). Our power was limited by the finite number of donors we were able to collect CSF from, the finite number of cells we could collect from each subject, and the limited transcript capture efficiency of the single-cell sequencing technology.^51^ The advent of biobank-scale single-cell datasets^26^ and efforts to pool and meta-analyze existing datasets will be essential for refining our understanding. One of the most notable features of our analysis is the considerable heterogeneity between individuals which is masked in *en masse* analyses. While it is necessary to pool data from many patients to draw general inferences about MS, the substantial variation between individuals merits caution when interpreting these results. Heterogeneity may be a feature of the noisy and sparse nature of single-cell data, may reflect true biological variation with confounders such as age, gender, and batch, or may reflect phenotypic characteristics of interest such as disease endophenotypes. Much larger datasets will be required to clarify these various possibilities. We aimed to mitigate issues of heterogeneity by using data from largely untreated subjects, considering our cohort in logical categories (non-inflammatory, infectious, non-infectious inflammatory, and MS), and careful inspection for and adjustment of batch effects.

While CSF is a useful and accessible tissue for understanding immune mechanisms in MS, it is a dynamic tissue which is unlikely to provide a perfect representation of events occurring within the brain, meninges, and draining cervical lymph nodes. Single-cell studies of brain and meninges are increasingly yielding novel disease insights, and it will be important to understand the extent to which single-cell examination of the CSF can provide an accurate readout of events in the brain and cord.^52^^,^^53^ Our data are a cross-sectional snapshot of a dynamic disease process. While a strength of our study is the largely untreated cohort of patients early in their disease course, there are likely to be valuable insights gained from performing these studies longitudinally to understand the impact of ongoing dynamic changes in the CSF on disease course, and control for important external influences—such as age, environmental factors, and disease-modifying treatment.

### Concluding remarks

We have presented the most comprehensive description to date of the transcriptomic and clonal landscape of CSF, shedding light on how this dynamic immune compartment is altered in inflammatory and infectious conditions. Our findings provide insights into the nature of CNS immunity in health and disease, shed light on disease pathomechanisms of relevance to other autoimmune diseases, and may suggest plausible targets for drug repurposing.

## Resource availability

### Lead contact

Queries relating to this manuscript should be directed to the lead contact, Prof. Bernhard Hemmer (hemmer@tum.de).

### Materials availability

The biosamples analyzed in this study are not available to other researchers.

### Data and code availability

• Single-cell RNA sequencing data have been deposited at the European Genome-Phenome Archive (EGA), which will be publicly available soon. Accession numbers are listed in the key resources table.
• All code used in this study is available on GitHub at https://github.com/benjacobs123456/cam_eu_scRNAseq.
• Any additional information required to reanalyze the data reported in this paper is available from the lead contact on request. The authors will share all data they are able to within the constraints of information governance rules and ethical regulations.

## Acknowledgments

This work was supported by funding from the UK Multiple Sclerosis Society (grant reference 99) and the 10.13039/100010661European Union’s Horizon 2020 Research and Innovation Funding Programme (EU RIA 733161) to MultipleMS. We acknowledge support from the 10.13039/501100000272National Institute for Health Research (NIHR) Cambridge Biomedical Research Centre, United Kingdom. We would like to thank the patients who have contributed to this study. B.M.J. is funded by an MRC Clinical Research Training Fellowship co-funded by the UK MS Society (MR/V028766/1). B.H. received funding for the study by the European Union's Horizon 2020 Research and Innovation Program (grants MultipleMS, EU RIA 733161, and WISDOM, EU ID: 101137154), the 10.13039/501100001659Deutsche Forschungsgemeinschaft (DFG, German Research Foundation) under Germany’s Excellence Strategy within the framework of the Munich Cluster for Systems Neurology (EXC 2145 SyNergy—ID 390857198), and the CLINSPECT-M consortium funded by the Bundesministerium für Bildung und Forschung (10.13039/501100002347BMBF), Germany. The Biobank of the Department of Neurology as part of the Joint Biobank Munich in the framework of the German Biobank Node supported the study.

## Author contributions

S.S., B.H., F.H., and C.G. recruited patients for the study. S.R.K. and M.B. performed the laboratory work. B.M.J. and C.G. led the data analysis and wrote the first draft of the manuscript. A.P. contributed to data analysis. M.B., S.S., and B.H. conceived the study and provided overall supervision and intellectual oversight. All authors were involved in critical review of the manuscript and take full intellectual ownership of its final contents.

## Declaration of interests

The authors declare no competing interests.

## STAR★Methods

### Key resources table

**Table:** 

REAGENT or RESOURCE	SOURCE	IDENTIFIER
**Biological samples**		

Peripheral blood mononuclear cells and cerebrospinal fluid samples from patients with MS and other neurological diseases	University of Cambridge & Technical University Munich	N/A

**Critical commercial assays**		

Chromium 5′ single-cell immune cell profiling	10X Genomics	https://www.10xgenomics.com/products/
Chromium 5′ V(D)J immune repertoire profiling	10X Genomics	https://www.10xgenomics.com/products/
Illumina NovaSeq	Illumina	https://emea.illumina.com/systems/sequencing-platforms/novaseq.html
Illumina HiSeq	Illumina	https://emea.support.illumina.com/sequencing/sequencing_instruments/hiseq_2500.html
Illumina Global Screening Array version 3	Illumina	https://emea.illumina.com/products/by-type/microarray-kits/infinium-global-screening.html

**Deposited data**		

Single-cell RNA sequencing data from MS patients and other neurological disease controls	This manuscript	EGA accession ID: EGAS50000000739; Technical University of Munich data: EGAS00001007954

**Software and algorithms**		

R programming language v 4.0.3, v 4.1.0, and v 4.2.2	The R project for statistical computing	https://www.r-project.org/
Seurat v 4.3	^54^^,^^55^^,^^56^^,^^57^	https://satijalab.org/seurat/
SoupX v 1.6.2	Young et al.^58^	https://github.com/constantAmateur/SoupX
DoubletFinder v 2.0.3	McGinnis et al.^59^	https://github.com/chris-mcginnis-ucsf/DoubletFinder
Harmony v 0.1.1	Korsunsky et al.^60^	https://github.com/immunogenomics/harmony
CellTypist v 1.6	Domínguez Conde et al.^61^	https://www.celltypist.org/
edgeR v 3.3.2	Robinson et al.^62^ Chen et al.^63^	https://bioconductor.org/packages/release/bioc/html/edgeR.html
Dandelion 0.3.2	Suo et al.^64^	https://github.com/zktuong/dandelion
CellRanger v 5.0.0	10X Genomics	https://www.10xgenomics.com/support/software/cell-ranger/latest
Vireo	Huang et al.^65^	https://github.com/single-cell-genetics/vireo
Cellsnp-lite	Huang et al.^66^	https://github.com/single-cell-genetics/cellsnp-lite
TOPMed-r2 imputation server	Taliun et al.^67^	https://imputation.biodatacatalyst.nhlbi.nih.gov/#! (Note that r2 is now not available and has been updated to r3).
PLINK versions 1.9 and 2	Chang et al.^68^	https://www.cog-genomics.org/plink/
Immunarch	Popov et al.^69^	https://immunarch.com

### Experimental model and study participant details

Two cohorts of individuals were recruited for the study, one from Cambridge University Hospitals trust in the UK and the other from the Technical University of Munich in Germany.

#### Cambridge cohort

The UK cohort was recruited through the Cambridge University Hospitals trust neurology department’s programmed investigation unit. Three groups of subjects were recruited: Patients with clinically-definite MS (as diagnosed by the neurologists in the department in line with the revised McDonald criteria^70^), patients with non-inflammatory neurological disorders, and patients with non-MS inflammatory disorders of the central and/or peripheral nervous systems. Details of included participants are shown in the supplement (Table S3). To verify the accuracy of the diagnoses in each case, all notes were reviewed by a consultant neurologist blinded to the single-cell RNAseq results (SS). We excluded patients with MS on current treatment with natalizumab, as this confounds the interpretation of the CSF single cell results.^13^ For one patient with an OIND, samples were obtained at two separate time points. These data were combined and treated as a single sample. Patients with CIS but negative CSF oligoclonal bands were classified as OINDs. We also collected venous blood and extracted PBMCs from a subset of the cohort. The study was approved by South Central – Berkshire NRES Ethics Committee (15/SC/0087) and all subjects gave fully informed consent.

#### TUM cohort

PBMC and CSF were collected from the TUM neurology clinic. Patients with MS, infectious neurological disorders, other inflammatory neurological disorders, and non-inflammatory controls were recruited via the same clinic. All MS patients recruited from this cohort have MS according to the 2017 McDonald criteria. The characteristics of these subjects are shown in the supplement (Table S3). All participants gave informed, written consent to participate in the study. The study was approved by the ethical review board of the Technical University of Munich (54/21 S-KK).

### Method details

#### Single-cell RNA sequencing

Samples were processed separately at either the University of Cambridge or the Technical University of Munich. A small number of subjects contributed samples which were processed independently at both sites for cross validation (noted with an asterisk in Table S3). Examination of the single-cell data generated at the different sites did not reveal significant batch effects and so these data were combined.

#### Cambridge processing

PBMC were extracted from venous whole blood using Ficoll-Paque density gradient centrifugation. Due to the low concentration of cells in the CSF, each sample was first concentrated by centrifugation at 300*g* for 10 min and the supernatant removed. The isolated PBMCs and CSF cells were frozen in up to 1mL of 10% DMSO and X-VIVO 10 Serum-free Hematopoietic Cell Medium (Lonza). The cryopreserved cells were rapidly thawed in a 37°C water bath and serially diluted with X-VIVO 10 Serum-free Hematopoietic Cell Medium. A manual cell count was completed using a Neubauer Haemocytometer and cell viability assessed using Trypan Blue exclusion dye. The cell suspension was centrifuged at 300g for 10 min to further concentrate the sample to a final volume of 34μL ready to be loaded onto 10X Chromium Single Cell Controller. We applied droplet-based single-cell RNA sequencing to all PBMC and CSF samples using the chromium 10X 5′ Genomics solution using global primers (for 5′ gene expression) and V(D)J-specific-primers (for immunoglobulin/T cell receptor gene analysis). cDNA libraries were sequenced using either the NovaSeq or Illumina HiSeq. An overview of the experimental design is shown in the supplement (Figure S1).

#### TUM processing

Freshly frozen and well-stored cerebrospinal fluid (CSF) cells or peripheral blood mononuclear cells (PBMCs) were thawed briefly at 37°C and quickly transferred to 15-mL falcon tubes containing ice-cold wash buffer (2% fetal bovine serum (FBS) in 1× phosphate-buffered saline, PBS). The cells were then centrifuged at 350 × g for 7 min. After centrifugation, the PBMCs/CSF cells were resuspended in wash buffer, and fractions of the resuspended cells were used for cell counting and viability assessment. For pooling CSF cells from different samples, we used barcoded TotalSeq C anti-human hashtag antibodies (BioLegend). Both surface and hashtag antibody stainings were conducted following the manufacturer’s instructions. We performed single-cell RNA sequencing using the 10X Genomics platform with Chromium Single Cell 5′ Reagent Kits and v2 Chemistry Dual Index. All single-cell processing steps were carried out using the Chromium Controller and 10X gel bead 5′ kits (for 5′ gene expression) according to the manufacturer’s guidelines. In addition to gene expression libraries, we generated T cell receptor, B cell receptor, and cell surface protein libraries using 10X Genomics kits. All sample libraries were subsequently sequenced on either an Illumina NovaSeq S2 or S4 flow cell.

### Quantification and statistical analysis

#### Data processing and quality control

##### Alignment and processing of raw reads

Raw sequencing output (fastq files) was processed using Cell Ranger v5.0.0. The Cell Ranger pipeline implements demultiplexing, alignment to the reference genome (hg38), and barcode counting.

##### Donor demultiplexing

To distinguish the donor of origin for cells run in multiplex, we used a combination of Vireo and CellSNPlite. CellSNPlite was used to infer SNP genotypes from scRNAseq data, and Vireo was then used on the inferred genotypes to infer the likely donor of origin for each cell.^65^^,^^66^ For the sample processed at TUM we additionally used hashtag antibody and the Seurat^54^ *HTODemux* function for demultiplexing.

##### Ambient RNA removal

Following initial quality control and alignment in Cell Ranger, data were loaded into SoupX program using the ‘load10x’ function. Quality control steps were performed individually for each batch prior to integration. We used SoupX^58^ to remove ambient RNA contamination from the count data. SoupX estimates the overall gene content of the ambient RNA ‘soup’ using empty droplets. Next, SoupX quantifies the average contamination rate by calculating the expression of marker genes in clusters which are expected to not express the gene - any detectable counts are therefore presumed to represent contamination by the soup. We experimented with three methods for estimating the contamination fraction - the automatic method, which calculates cluster-specific genes based on an information theoretic metric - manual curation, whereby cluster-specific genes are pre-specified based on biological knowledge, and a brute force method whereby the global contamination rate is set at an arbitrary threshold. For all of these methods, the clusters pre-computed by Cell Ranger were used as inputs. We achieved optimum results with the manual method by pre-specifying a list of hemoglobin and immunoglobulin genes, which are expected to be highly expressed in red blood cells and B cells respectively, and should be specific to these cell types. This contamination rate was then used to adjust the raw count data, and the corrected counts were transferred into a Seurat object.

##### Filtering in seurat and doublet detection

The percentage of reads mapping to mitochondrial genes was calculated using the ‘PercentageFeatureSet’ function in Seurat. We excluded cells with ≥ 10% mitochondrial reads, and with <100 RNA molecules per cell. We removed red blood cells by filtering out cells for which >1% of reads mapped to *HBA1*, *HBA2*, or *HBB*. Next, to detect homotypic doublets - cell doublets where both cells originate from the same donor - we used DoubletFinder.^59^ Initial clustering was first performed using SCTransform, the first 10 Principal Components, and the default graph-based clustering methods in Seurat (‘FindNeighbours’ and ‘FindClusters’). We assumed a 7.5% rate of doublet formation and ran DoubletFinder with default parameters. To detect heterotypic doublets in the multiplexed batches (i.e., doublets where the cells originate from different donors) we ran Vireo and CellSNP (see above). We excluded all cells called as a doublet or ‘unassigned’, i.e., where no donor could be confidently assigned.

##### Normalisation

Following correction for ambient RNA contamination, removal of homotypic and heterotypic doublets, and removal of low-quality cells, we normalised counts using SCTransform.^71^ SCTransform fits a negative binomial regression model to the count data for each gene separately, regressing out the overall sequencing depth, and then regularises the parameters over all genes. We used this procedure to regress out the mitochondrial gene percentage for each cell. The residuals from this regression model reflect the corrected counts for each gene. We used the ‘glmGamPoi’ plugin to improve speed.^72^

##### Integration across batches

Following batch-level quality control and exclusion of poor quality batches, we integrated data across all batches to facilitate downstream analysis. We merged all datasets using the ‘merge’ function in Seurat. Next, we selected the 10,000 most variable genes across all datasets using the ‘SelectIntegrationFeatures’ function. We computed the first 50 Principal Components using these variable genes and the SCTransform-corrected count data. We used Harmony to minimise the effects of batch on cluster assignment^60^ - Harmony is an iterative algorithm which calculates batch-specific correction factors and adjusts each cell’s PC embeddings by these factors. The union of variable genes across batches resulted in 4,083 variable genes used for dimension reduction.

#### Clustering and cell-type annotation

To define cell types within the dataset, we performed unsupervised clustering using the default graph-based clustering methods in Seurat. We used the first 50 Harmony-adjusted PCs as inputs for the FindNeighbours and FindClusters functions. To explore which clustering parameters yielded the most biologically-meaningful clusters, we examined effects of modifying either the number of Harmony-adjusted PCs or the clustering resolution. Ultimately we used the first 50 PCs and a resolution of 2.5 for downstream analyses. As sensitivity analyses, we repeated the clustering step using a range of resolution parameters and PCs.

To determine the identity of the observed clusters, we used two approaches. First, we calculated the top cluster-defining genes for each cluster using the ‘FindAllMarkers’ function with default parameters, which implements the Wilcoxon Rank-Sum test to identify differentially-expressed genes between the index cluster and all other cells. These cluster biomarkers were used alongside canonical markers genes to define known cell types. Second, we compared these manual annotations with automatic cell annotations calculated using CellTypist and the SingleR^73^ package. For SingleR annotations, we used the Blueprint/ENCODE,^74^^,^^75^ Database of Immune Cell Expression (DICE),^34^ Human Primary Cell Atlas (HPCA),^76^ and Monaco Immune ref. ^77^ expression datasets accessed via the celldex R package.

#### Differential expression

To determine whether gene expression differed between CSF and blood, or between MS and control, we used pseudobulk methods for estimating differential expression. Pseudobulk methods pool gene counts over all cells within an experimental condition, rather than treating each individual cell as a replicate. These methods provide better control of type I and type 2 error than dedicated single-cell methods.^78^^,^^79^ Differential expression between CSF and PBMCs and between disease cohorts was assessed using negative binomial models implemented in edgeR.^62^ Raw, non-normalised, SoupX-adjusted counts were first aggregated across cells per cluster, per body fluid and per donor. We then removed groups (i.e., pseudobulks for each donor, source, and cell type) where the overall cell count contributing to the pseudobulk was <10. This requirement for 10 cells was reduced to 2 cells for the clonal B cell analysis due to low cell numbers. Next, genes with low overall pseudobulk counts were removed from the analysis using the ‘FilterByExpr’ function in edgeR. Pseudobulk counts were then normalised using the trimmed mean of M values method.^80^ This method calculates the mean log fold change in the relative abundance of gene counts between samples for genes expected to be invariant between samples, and is thus based on the assumption that the majority of genes are not differentially expressed between samples/conditions.^80^ We used the quasi-likelihood F test implemented in edgeR’s ‘glmQLFTest’ to evaluate the statistical significance of differentially-expessed genes.^63^ All models were adjusted for age and gender.

#### Differential cell type abundance

Differences in the relative abundance of cell types between CSF/PBMC and between MS/controls were tested using negative binomial models in edgeR. For the primary analyses we adjusted for age and gender. Reported changes represent log2 fold changes in the proportion of the cell type between conditions. Changes were assessed using the quasi-likelihood test in edgeR. Absolute counts of each cell type were normalised to the log of the total number of cells within the same compartment of the same donor. Statistical significance was determined using a False Discovery Rate (FDR) of 5%.

#### Gene set enrichment analysis and pathway analysis

Gene set enrichment analysis (GSEA) of differentially-expressed genes was performed using the Fast Gene Set Enrichment Analysis (fgsea)^81^ R package. Fgsea compares the rank of each gene in the test set vs. the reference gene set to calculate an enrichment score. It then calculates an empirical *p* value for the enrichment score by sampling random gene sets of equal size. For the reference gene sets we used the Hallmark pathways downloaded from the MSigDB^82^ via the MSigDBr R package.^83^ Fgsea was run using a minimum gene set size of 10, Hallmark gene sets, and 10,000 permutations. We reported pathways with an FDR of 1% - controlling for all the pathways tested within each cell type within each specific comparison. Weighted pathway analyses and transcription factor activity analyses were conducted using the PROGENy^84^ and DoRothEA^20^ resources respectively, implemented in DecoupleR.^19^

#### Lymphocyte receptor repertoire analysis

We re-processed 5′-VDJseq contigs using the Immcantation^85^ pipeline implemented in Dandelion.^64^^,^^86^ Briefly, this procedure performs three quality control steps: reannotation of *IGHC* constant region calls, V(D)J gene reannotation, and reassignment of V gene segment alleles using germline information. We restricted our B cell dataset to those cells meeting the following criteria: present in the VDJ-seq dataset; expresses exactly one heavy chain and one light chain contig; each contig passes quality control for read quality; doublets; productive chains. Clonal B cells were defined as cells which shared identical heavy and light chains, with identical length CDR3 sequences, and with CDR3 similarity >85% (as quantified by the length-normalised Hamming distance).

TCR sequences were also preprocessed using Dandelion. Quality control procedures were similar to those for BCRs: we excluded cells not classified as T cells or absent from the gene expression dataset, cells without alpha and beta chains, cells with low quality or non-productive contigs, and doublets. We excluded gamma-delta T cells, although these were few in number due to the nature of the primers used in the library preparation (which target the constant region of the *TRA* and *TRB* chains). Clones were defined using similar criteria to the BCR definition with a stricter CDR3 similarity criterion (100%). To prevent loss of large numbers of cells with high-quality *TRB* data but no *TRA* data, we defined clonal groups based solely on TCR beta chains.

To determine the specificity of TCRs detected in our dataset, we combined our data with a public database of experimentally-determined TCR specificity downloaded from VDJ-DB via the Immunarch R package.^69^ We matched TCRs based on two approaches: first, stipulating only that the CDR3 amino acid sequences were identical; second, stipulating both matching CDR3 sequences and identical TRBV gene usage. Empirical *p* values for enrichment were calculated by resampling the dataset with replacement 1000 times and comparing the proportion of TCRs specific for each epitope in MS vs. each control cohort in each permutation. Empirical one-tailed *p* values (for the alternate hypothesis that the TCR was enriched in MS) were calculated as 1 - proportion of trials in which MS was enriched.

#### Single-cell eQTL mapping

##### Genotype data quality control and imputation

Genotype quality control was performed in using PLINKv1.9 or 2.0.^68^ The 80 samples from the Cambridge cohort were genotyped using the Illumina Infinium Global Screening Array-24 version 3 (GSAv3) genotyping array and quality controlled prior to imputation as follows: We removed individuals with high missingness (>10%, *n* = 0 removed) and SNPs with low MAF (<0.05), deviation from HWE at *P* < 1x10^−5^, or high missingness (>10%). Imputation of these samples was performed using the TOPMed-r2 panel via the TOPMed imputation server.^67^^,^^87^ After imputation, we removed all variants with an INFO score below 0.7 and a minor allele frequency below 0.001. The 127 samples from the TUM cohort were genotyped on the same array (Illumina GSAv3) as part of larger cohorts. Prior to imputation, we performed quality control (QC) where we removed variants out of Hardy-Weinberg equilibrium (*p* < 1 x10^−6^), with minor allele frequency (MAF) < 0.001 or with missingness greater than 2%. We further removed individuals with sample missingness greater than 4.5%, individuals with mismatch between the genetic sex and the reported gender, with excess heterozygosity of more than 5 standard deviations (SD) from the sample mean, and population outliers in principle component space. Phasing was performed using SHAPEIT2 (version 2.r837)^88^ with standard settings and imputation was performed with IMPUTE2 (version 2.3.2)^89^ to the 1000 Genomes Phase 3 reference. After imputation, we removed all variants with an INFO score below 0.7 and a minor allele frequency below 0.001.

##### Merging and joint quality control

Genotyped and imputed genetic data from the TUM cohort was mapped to the Genome Reference Consortium Human Build 38 (GRCh38) and merged with the Cambridge genotype data. After merging, we removed variants with a MAF <0.01 in either of our two datasets or the 1000 Genomes reference data, variants with a MAF difference of >0.2 between the CAM and the TUM datasets, variants with a deviation of the MAF from the 1000 Genomes reference data MAF of >0.2 as well as strand-ambiguous (palindromic) SNPs with a MAF between 0.4 and 0.6.

We removed individuals with a missingness rate >2% (*n* = 0), with excess heterozygosity of more than 5 SD from the sample mean (*n* = 0), relatives (*n* = 1, determined using KING kinship coefficients calculated with plink –king-cutoff with a threshold of 0.125), and population outliers (*n* = 0) with a distance in the first 8 principal components of more than 4 SD from the mean. These QC steps were performed using a set of genetic variants with MAF >0.05, genotyping rate >0.02, pairwise linkage disequilibrium (LD) < 0.2 and an HWE test *p* value < 1x10^−6^). For the determination of population outliers we further remove the MHC region on chromosome 6 (25Mbp-35Mbp) and the INV8 region (chromosome 8, 7-13Mbp). Finally, we removed insertions and deletions and variants with a genotype missing rate >2% and an HWE test *p* value < 1x10^−3^. After QC, 173 individuals and 5,018,132 variants were left for analysis.

##### Gene expression data quality control and preparation for eQTL mapping

Gene expression data quality control was performed for CSF cells and PBMCs separately. We used SCTransform^71^^,^^90^ to normalize counts on a single-cell level which we performed on each batch separately and then calculated the mean expression per individual and cell type. We removed genes that were expressed in less than 2% of the cells. For each cell type we further removed individuals with less than 5 cells of the specific cell type as well as genes that were expressed in the cell type in less than 20% of the individuals from the analysis. For eQTL analysis, we selected genes that have been prioritised as potential causal genes for MS risk^29^ or which showed differential expression in CSF compared to PBMC in our study in T cells or B cells (with a Bonferroni adjusted *p*-value <0.05 and an absolute logFC >0.5).

##### eQTL analysis

In the primary analysis, we performed single-cell eQTL analysis separately for the three main cell types - B cells, CD4^+^ T cells and CD8^+^ T cells. We tested for association between normalised gene expression and all SNPs within +/− 500KB of the start and endpoints of the gene. Association testing was conducted using linear models in PLINK2.^68^ To adjust for genetic population structure we calculated PCs with PLINK using an LD-pruned set of variants with MAF>0.05 and HWE p < 1x10^−3^ and added the first 5 PCs to the regression models. To adjust for experimental batch effects we performed PCA using the R function *prcomp* on the aggregated expression values for each donor for all genes tested and included the first four principal components in our models. We further adjusted our regression models for age and gender. *P* values were adjusted using a false discovery test across all tests performed. For eQTLS with an FDR adjusted *p* < 0.1 we determined whether these associations had previously been described using the available datasets from the GTEX consortium,^27^ the eQTLGen project^28^ or a recent large single cell eQTL analysis on peripheral blood mononuclear cells^26^ and performed permutation analysis (up to 1,000,000 permutations) for previously unreported associations. To compare effect sizes across cell types, we further performed eQTL analysis on the remaining 8 cell types in secondary analyses.

##### Compartment-specific effects

We determined the correlation between effect sizes in different cell types by considering MS CSF eQTLs significant at an FDR of <10%. To formally test for effect size heterogeneity, we calculated heterogeneity *p* values by comparing Z scores for heterogeneity across different cell types:

$$Z_{Heterogeneity}=\frac{\beta_{1}-\;\beta_{2}\;}{\sqrt{{SE}_{1}^{2}+{SE}_{2}^{2}}}$$

We considered eQTL effects to show evidence of CSF specificity if they satisfied the following conditions.

• FDR-adjusted heterogeneity *p*-value <0.05, AND
• FDR adjusted association *p*-value <0.1 AND association *p* (unadjusted) > 0.01 in all tested PBMC cell types AND
• no previous report (even at nominal significance with *p* < 0.05) of the eQTL^26^^,^^28^^,^^32^^,^^33^ AND
• permutation *p*-value below the maximal *p*-value reaching a studywide FDR <0.1 (if not previously reported)

##### Colocalization analysis

To assess overlap between MS susceptibility GWAS hits and eQTLs, we performed statistical colocalization under the single causal variant assumption using the Coloc R package.^91^ By inferring Bayes factors from GWAS beta estimates and standard errors, Coloc evaluates the posterior probability, at each variant, that the variant is the causal variant underlying both traits. We performed colocalization analyses for each of the identified loci with an nominally significant eQTL (*p* < 0.0001) and assumed a single causal variant within the window tested (lead eQTL SNP +/− 200KB). MS GWAS summary statistics were obtained from the discovery-stage meta-analysis of the IMSGC 2019 susceptibility GWAS^29^ and were converted to hg38 using the LiftOver command line tool. A posterior probability for colocalization >70% was used as the threshold for the identification of colocalized association signals.

#### Power considerations for clonal detection

Given that the total number of B cell clonotypes in the body (3.5x10^10^) is very nearly the same as the total number of B cells (10^11^)^92^ it is unsurprising that modeling indicates that the vast majority of receptor sequences are only carried by one or a very few cells. At the other end of the frequency distribution modeling predicts marked skewing with the 20 largest clones typically account for almost 2% of all B cells.^93^ Because processes such as germline gene usage and clonal selection are non-random, there are biases in favor of certain receptor sequences.^94^ As a result, despite the fact that the total number of clonotypes carried by an individual is orders of magnitude less than the number of potentially possible sequences,^95^ these biases generate so-called “public” sequences that are carried by a high proportion of individuals. These shared public sequences typically make up few percent of any given individuals’ repertoire^96^; for the T cell receptor the proportion of sequences that are public is somewhat higher.^94^ The probability of seeing two cells from the same clone in any given individual is not only dependent upon the size of the clone and the number of cells sampled, but is also profoundly influenced by the partitioning of the immune system and the localized nature of clonal expansion, factors which together result in a non-uniform distribution of the daughter cells from any clone. By its very nature the blood-brain barrier tends to isolate the intrathecal part of the immune system from the rest of the immune system.^97^ As a result, a clone generated intrathecally is essentially only part of the intrathecal B cell repertoire, rather than being distributed throughout the entire immune system. In healthy individuals, the brain typically has a volume of 1450mL and a B-cell concentration of 200 cells per mL so that the organ will on average contain a total of approximately 300,000 B-cells. Likewise, the CSF, which has a volume of 150mL and an average white cell count of 1 per mm^3^, only 1% of which are B cells, will typically contain around 1,500 B cells. This number is much smaller than the number of cells in the brain and thus necessarily only partially reflects the intrathecal B cell repertoire. In our study we aimed to assay approximately 1000 CSF cells from each individual, a number which in healthy individuals would on average be expected to include just 10 B cells.^98^ In this context our paradigm has essentially no power (<1%) to identify two or more cells from the same clone unless the clone is very large (constitutes >5% of all B cells in the CSF). In other words, in healthy (NIND) subjects our sampling strategy has no meaningful power to identify B cell clones. In the context of neuroinflammation however the proportion of B cells in the CSF is increased and thus more will be included within the sample of 1000 cells. If we suppose that an inflammatory reaction results in the generation of 15 clones each of 100 cells then the total number of B cells in the CSF will be doubled so our sample of 1000 cells will include 20 B cells. Furthermore, each clone will represent 3% of the CSF B cells. In this situation we would still only have modest power to identify two or more cells from any particular clone (2%) but will have very high power (>96%) to identify daughter cells from at least one clone. Given that the average person has 5 L of blood which contains an average of 157 B cells per microlitre^99^ there are a total of 785 million B cells in the peripheral circulation. In the periphery therefore the equivalent clonal expansion would represent a trivial fraction of the total and there would be no meaningful power to detect two or more cells from the same clone. The identification of a B cell clone thus indicates that either the clone is extremely large or more likely are from a clone that has been generated close to or within the CSF space. The accepted dogma that the appearance of expanded B cells in the CSF is a feature of neuroinflammation is in fact a stoichiometric consequence of inflammation occurring within a space where there is normally only a very limited number of resident B cells. The same reaction within the periphery would be undetected as it would be diluted by the larger representation of the B cell repertoire in the periphery.

## Supplemental information

Document S1. Figures S1–S14 and Tables S11–S13

Table S1. Numbers of cells analyzed by compartment and disease status, related to Figure 1 and Table 1

Table S2. Numbers of donors according to site, compartment, and disease state, related to Figure 1 and Table 1

Table S3. Demographics of included participants and cell numbers per sample, related to Figure 1 and Table 2

Table S4. Cluster annotations at “low” and “high” levels including CellTypist consensus annotations and cluster-defining genes, related to Figure 1 and Table 2

Table S5. Differential abundance results, related to Figure 1 and Table 2

Table S6. Differential expression results, related to Figure 2

Table S7. Differential expression results comparing expanded memory B cells in MS CSF with non-expanded memory B cells in MS CSF, related to Figure 3

Table S8. Differential abundance of T cell subsets in CSF versus PBMC across all disease cohorts, related to Figure 4

Table S9. Seemingly CSF-specific eQTLs, related to Figure 5

Table S10. Colocalization between MS risk signals and eQTLs, related to Figure 5

Document S2. Article plus supplemental information
