# Supplementary material for: Single-cell analysis of cerebrospinal fluid reveals common features of neuroinflammation
Source: Cell Rep Med. 2024 Dec 20;6(1):101733. doi: 10.1016/j.xcrm.2024.101733 (PMC11866449; doi:10.1016/j.xcrm.2024.101733)
Supplement: Document S1. Figures S1–S14 and Tables S11–S13 [file mmc1.pdf]

Cell Reports Medicine, Volume 6

Supplemental information

Single-cell analysis of cerebrospinal fluid reveals common features of neuroinflammation

Benjamin M. Jacobs, Christiane Gasperi, Sudhakar Reddy Kalluri, Raghda Al-Najjar, Mollie O. McKeon, Jonathan Else, Albert Pukaj, Friederike Held, Stephen Sawcer, Maria Ban, and Bernhard Hemmer

Supplementary materials for "Single cell analysis of cerebrospinal fluid reveals common features of neuroinflammation"

Authors:
Benjamin M Jacobs* [1,2], Christiane Gasperi* [3], Sudhakar Reddy Kalluri* [3], Raghda Al-Najjar [1], Mollie McKeon [1], Jonathan Else [1], Albert Pukaj [3], Friederike Held [3], Stephen Sawcer+ [1], Maria Ban+ [1], Bernhard Hemmer+ [3,4]
* equal contribution
+ joint corresponding authors

Table of contents

Experimental design 2

Preprocessing and quality control 2

Cell type annotation 3

Individual-level heterogeneity and disease phenotype 5

Mechanisms of B cell and antibody-secreting cell (ASC) recruitment to CSF 7

Differential expression & pathway analysis 8

B cell repertoire 9

TCR repertoire 13

Expression QTLs 13

References 16

Experimental design

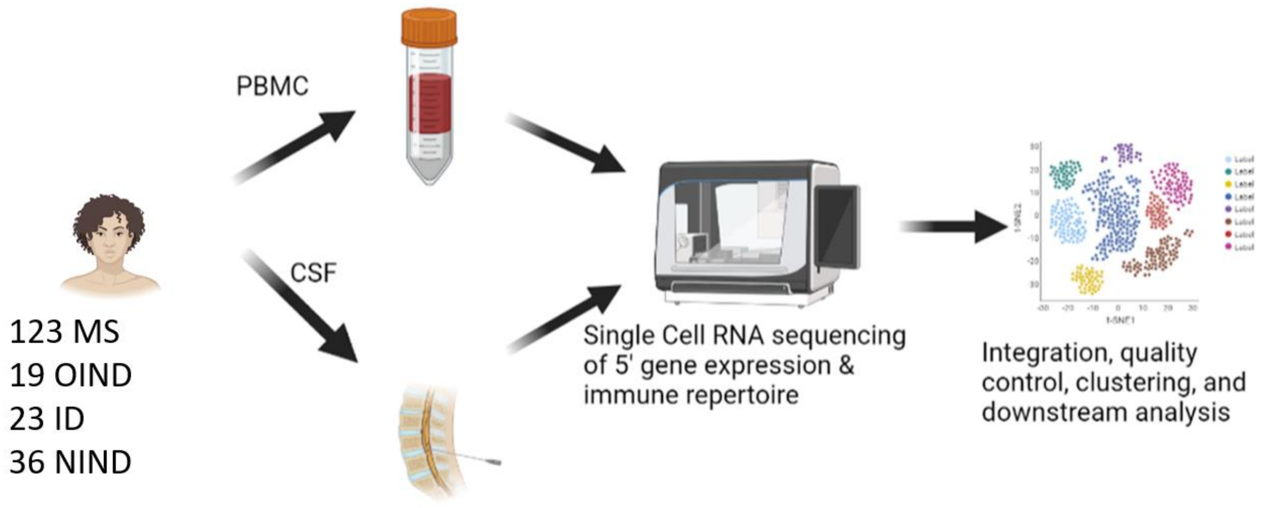

Supplementary figure 1: **Overview of experimental design, related to figure 1.**

Preprocessing and quality control

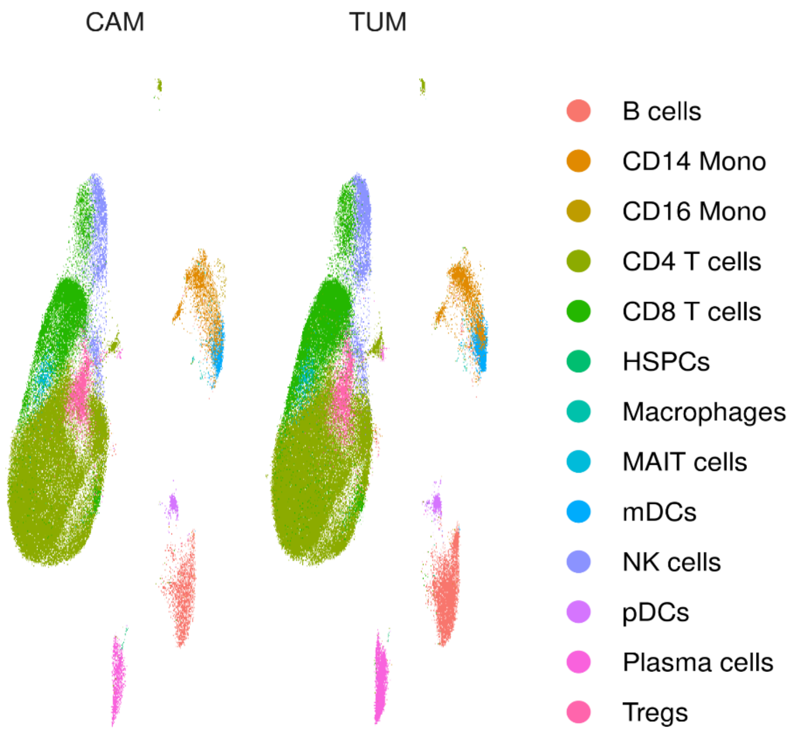

Supplementary figure 2: **Integration of datasets generated across two sites, related to figure 1.** UMAP plot showing single-cell data following integration, revealing no obvious batch effect due to processing site (CAM = Cambridge, TUM = Technical University Munich). Data are shown for the 6 individuals who were processed independently at each site and thereby duplicated. We attempted to mitigate batch effects using a workflow incorporating SCTransform normalisation and Harmony-based integration. Visual inspection of the gene expression data partitioned by processing site did not reveal obvious batch effects after integration.

Cell type annotation

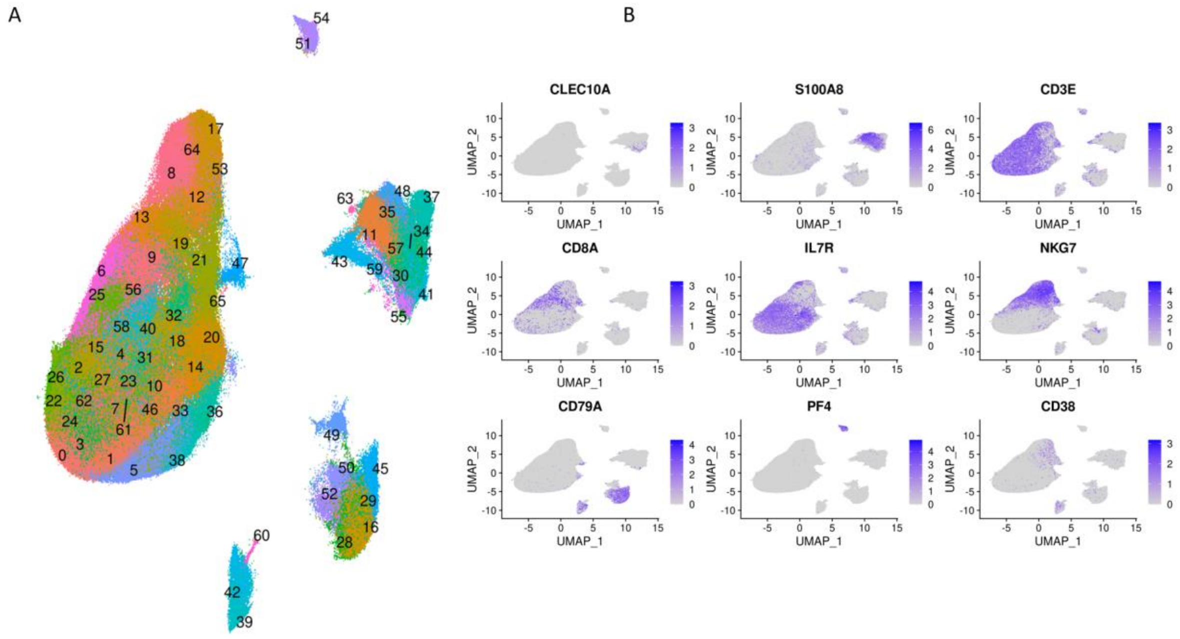

Supplementary figure 3: Cell type annotation, related to figure 1. A - joint clustering of CSF and PBMC samples following removal of low-abundance clusters, red blood cells, and platelets. B - expression of selected canonical markers used (among others) to annotate clusters with cell type labels. Cell type annotation was performed using canonical marker expression, reference annotation with CellTypist, and cluster-defining biomarkers.

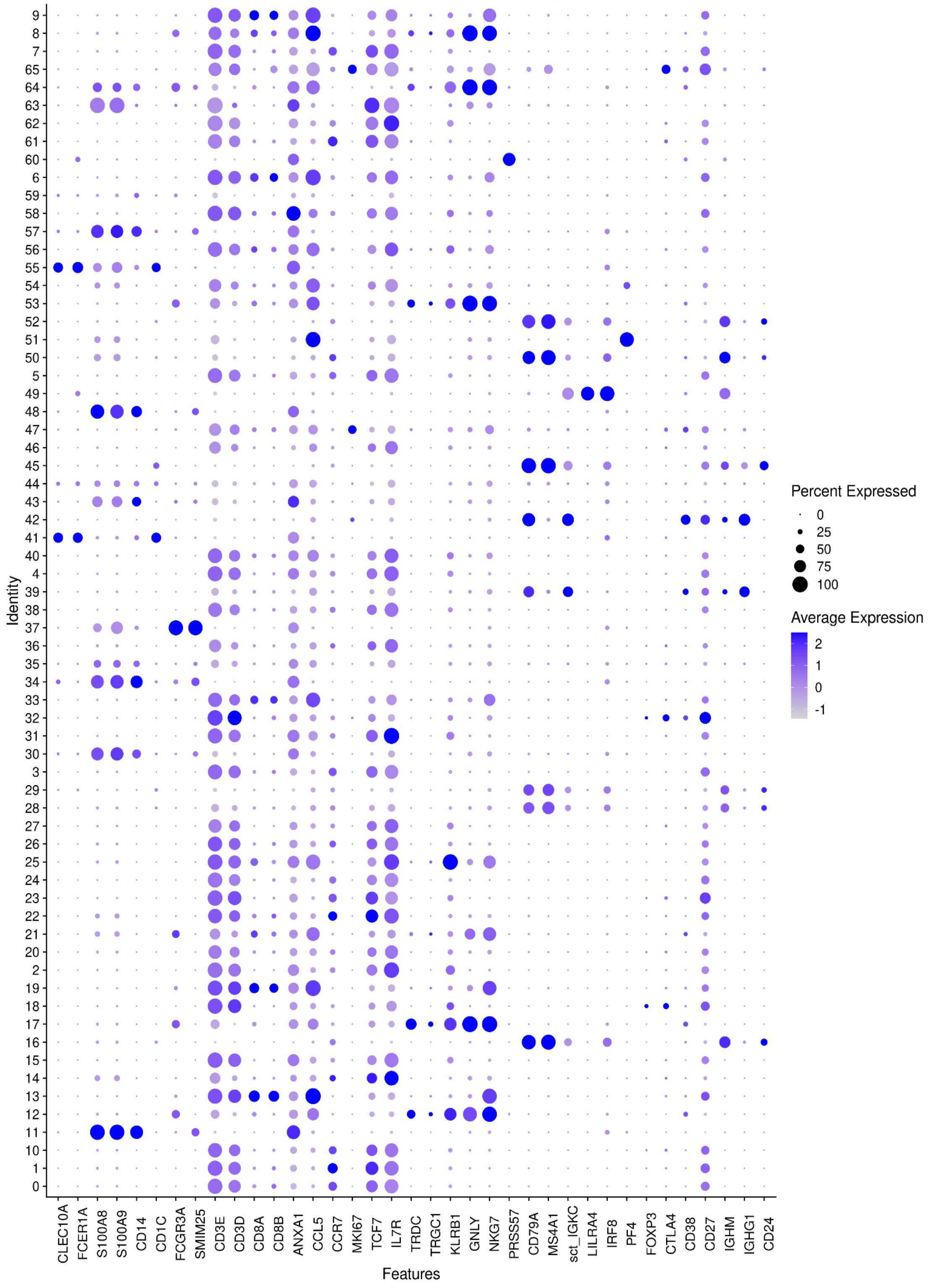

Supplementary figure 4: **Canonical marker expression within cell type clusters, related to figure 1.** Dot plot showing the expression of key canonical markers in each raw cluster. Cluster identities following annotation can be found in supplementary table 4. Expression of canonical markers was used to cross-reference cell type annotations. We grouped cells into the following high-level groups: CD4+ T cells (*CD3E,CD3D*, *IL7R*), CD8+ T cells (*CD3D*, *CD3E*, *CD8A*, *CD8B*), NK cells (*GNLY*, *NKG7*), regulatory T cells (Tregs; *CD3E*, *CD3D*, *FOXP3*, *CTLA4*), CD14+ classical monocytes (CD14+ Mono; *S100A8*, *S100A9*, *LYZ*), B cells (*CD19*, *CD20*, *CD79A*), Plasma cells (*CD19*, *CD27*, *CD38*, *IGKC*), Haematopoietic stem cells (HSCs; *SOX4*, *PRSS57*),

plasmacytoid dendritic cells (pDCs; *LILRA4*, *IRF8*), myeloid dendritic cells (mDCs; *FCER1A*, *CLEC10A*), CD16+ non-classical monocytes (CD16+ Mono; *S100A8, S100A9, SMIM25, FCGR3A/CD16*), and MAIT cells (*CD3D, CD3E, KLRB1)*.

Individual-level heterogeneity and disease phenotype

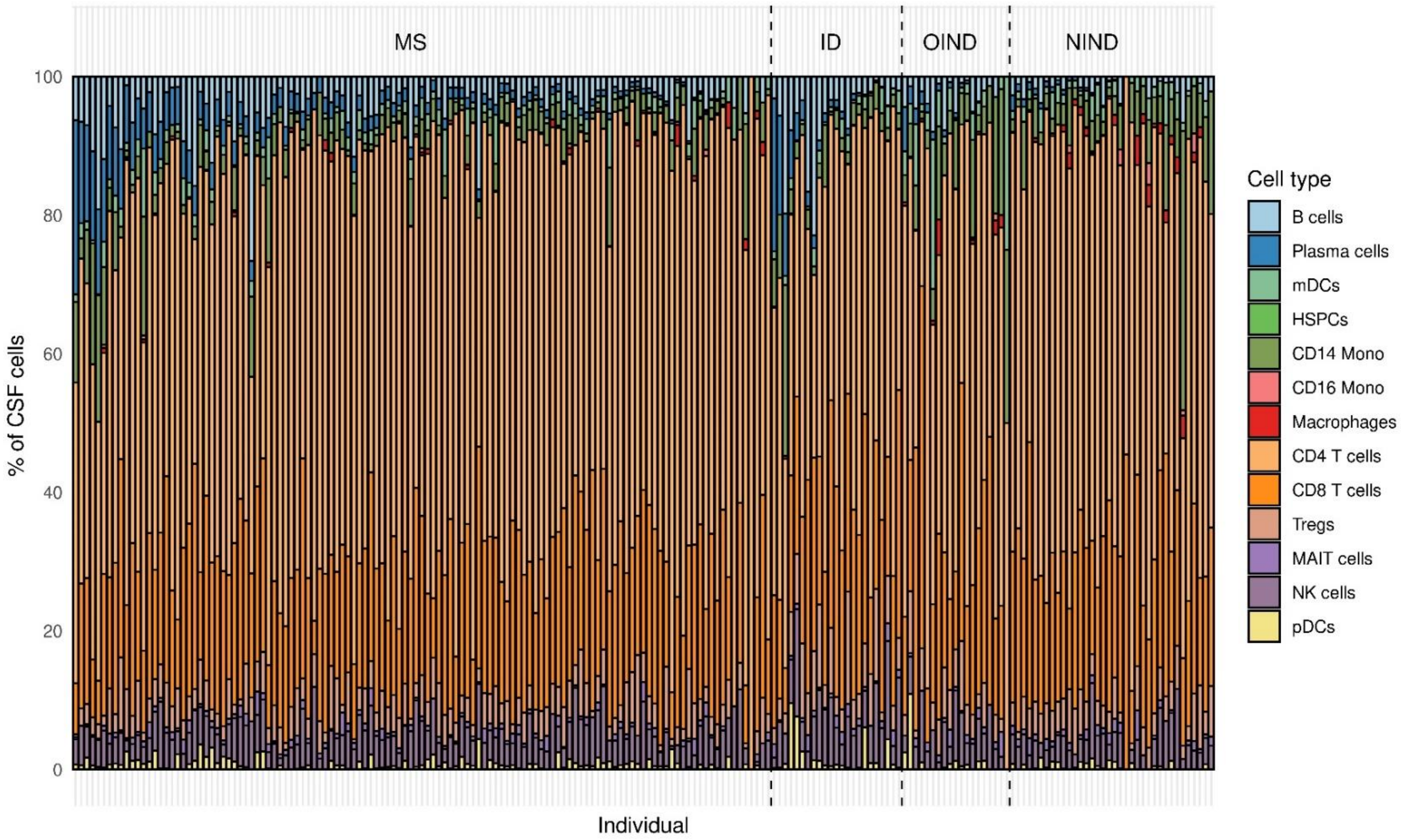

Supplementary figure 5: CSF and PBMC compositional heterogeneity between and within phenotype categories, related to figure 1. Individual-level cell type proportions in CSF samples divided by diagnostic category. We observed substantial heterogeneity in cell type proportions between individuals, even within disease cohorts. The proportion of CSF cell types per individual, categorised by disease status is shown in the plot. The y axis depicts the proportion of cells consisting of each cell type within the CSF sample compartment for each individual. Bars are stacked and sum to 1. Bars are coloured by cell type. Each bar on the x axis reflects an individual donor. Demographic details of participants included in this study are shown in table 1 and supplementary table 3. The initial cohort comprised 126 people with MS, 41 non-inflammatory neurological controls (NINDs), 19 other inflammatory neurological disease controls (OINDs), and 23 infectious neurological disease controls (IDs). From this cohort, we obtained CSF samples from 123 MS patients, 36 NINDs, 19 OINDs, and 23 IDs. PBMC samples were obtained from 76 MS patients, 28 NINDs, 12 OINDs, and 4 IDs. We excluded one OIND sample which had a very low cell count (<5 in CSF sample) despite adequate apparent sample quality and volume - sc_169_OIND, omitted from supplementary table 4). In addition, we excluded the MS patient who was on current treatment with natalizumab (sc_66_MS) due to the mechanism of action of this drug, which excludes leukocytes from the CSF and so distorts the compartment-specific changes due to the disease process. The age distribution, gender composition, and CSF oligoclonal band status of each of the four groups are shown in table 1. Most of the MS cohort had relapsing MS at the time of sampling (116 / 126, 92.1%) with the remainder having Primary Progressive MS or Secondary Progressive MS. The NIND group comprised a diverse cohort with diagnoses ranging from headache syndromes (largely migraine and idiopathic intracranial hypertension), suspected motor neuron disease, cerebrovascular disease, functional neurological disorders, non-inflammatory peripheral neuropathy, and idiopathic cranial neuropathies felt to be non-inflammatory in nature. The most common diagnosis in this group was a headache syndrome (23 /

40, 57.5%). The OIND cohort comprised systemic autoimmune diseases with CNS involvement (including sarcoid and SLE), Chronic Inflammatory Demyelinating Polyradiculoneuropathy, unspecified CNS inflammatory disorders under investigation (such as suspected CNS vasculitis and aseptic meningitis), and Clinically Isolated Syndrome (CIS) where the patient had not met diagnostic criteria for MS. The ID group comprised several cases of neuroborreliosis, acute VZV-associated facial neuropathy, acute infectious meningoencephalitis.

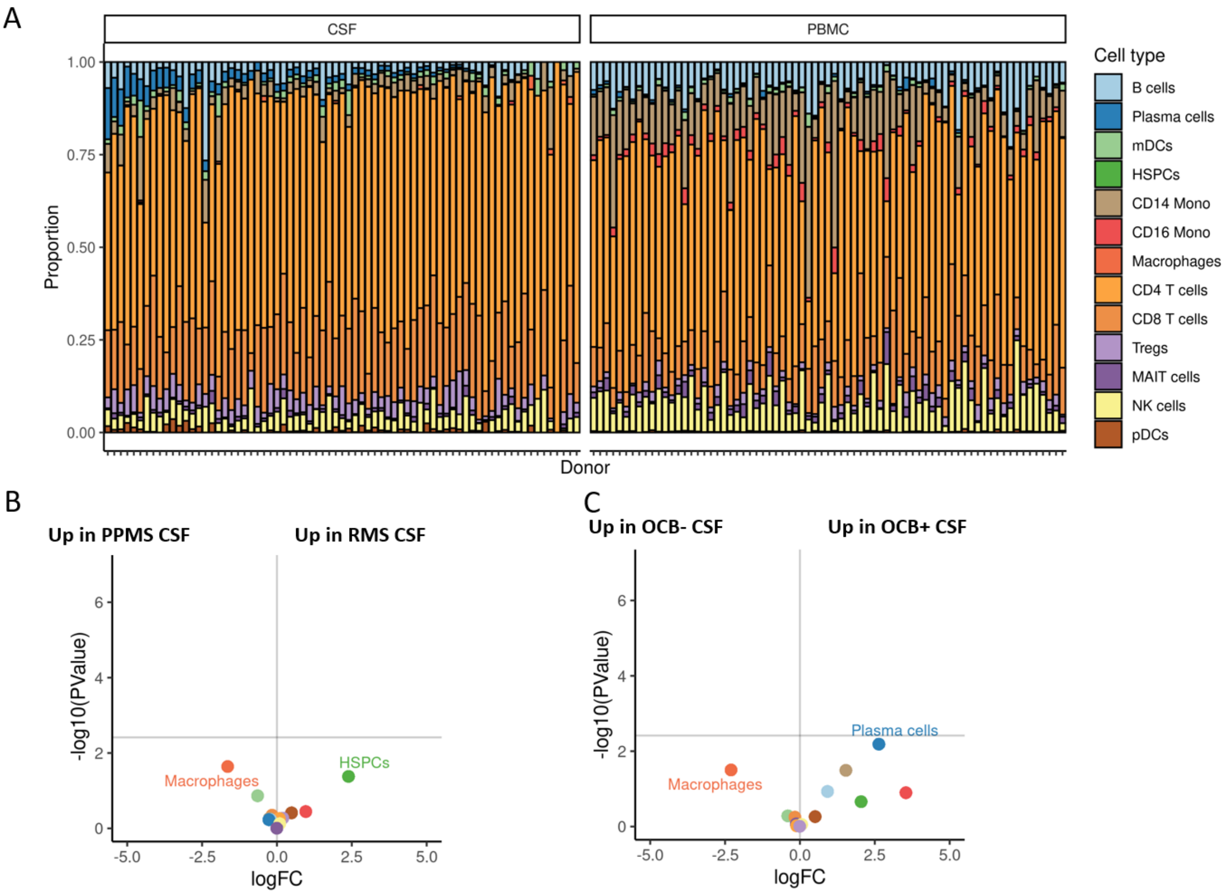

Supplementary figure 6: **CSF and PBMC compositional heterogeneity in relation to MS disease characteristics, related to figure 1.** To explore whether this heterogeneity was related to MS subtype or oligoclonal band (OCB) status, we compared CSF composition between relapse-onset MS and primary progressive MS, and between oligoclonal band positive and negative cases. We found minimal difference between MS subtypes, but found the expected suggestive increase of CSF plasma cells in OCB+ patients. A - cell type proportions in CSF and PBMC of MS patients. The y axis depicts the proportion of cells consisting of each cell type within the compartment for each individual. Bars are stacked and sum to 1. Bars are coloured by cell type. Each tick on the x axis reflects an individual donor. Only donors with both CSF and PBMC samples are shown. B - differential abundance volcano plot comparing the proportions of cell types in CSF between donors with relapse-onset MS (RMS) and primary progressive MS (PPMS). The horizontal line indicates the Bonferroni-corrected P value threshold (alpha = 0.05). The x axis shows the log fold change in cell type proportion between the two conditions. C - As per B but contrasting donors with (OCB+) and without (OCB-) detectable CSF oligoclonal bands.

Mechanisms of B cell and antibody-secreting cell (ASC) recruitment to CSF

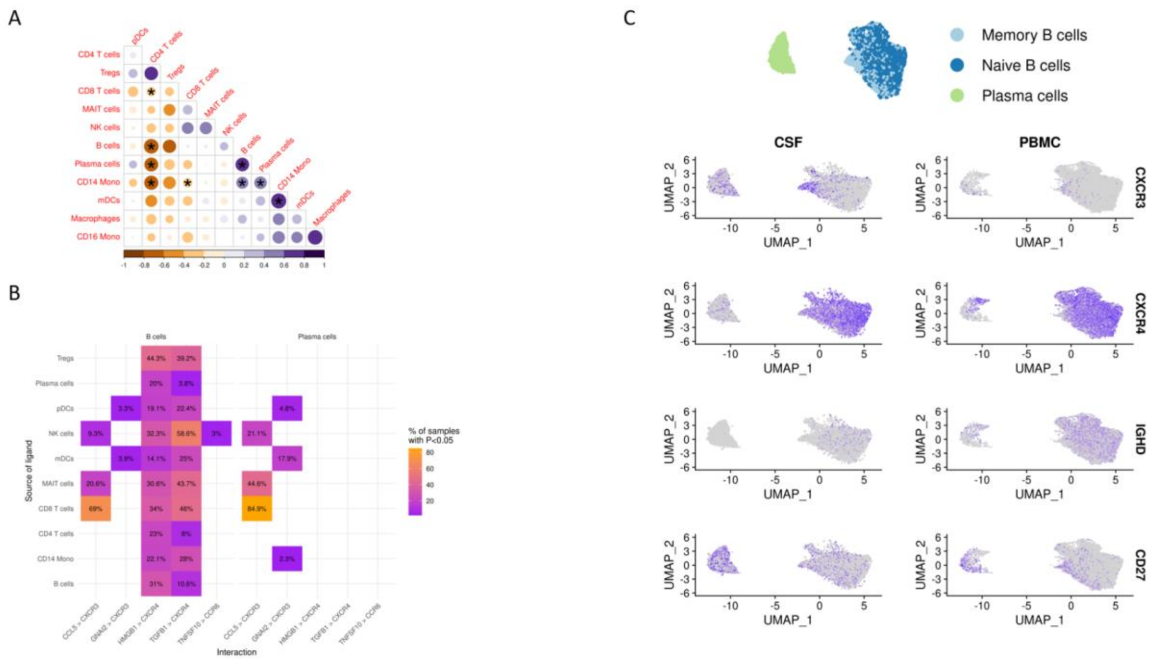

Supplementary figure 7: Putative ligand-receptor pairs facilitating B cell recruitment to the CSF, related to figure 1. In an attempt to identify the molecular drivers of B cell / ASC entry in to the CSF in MS, we examined the expression of chemokine receptors on B cells and ASCs in MS CSF along with their ligands using the Ligand-Receptor Analysis Framework (LIANA[1]). We ran LIANA on a per-sample basis and considered interactions achieving a P value of <0.01 in at least 50% of samples). We restricted the analysis to the receptor-ligand interactions with canonical chemokine receptors (downloaded from https://www.genenames.org/data/genegroup/#!/group/189), so that in total 67 distinct ligand-receptor pairs were considered. In ASCs we observed strong evidence for putative interaction between *CCL5* expressed by CD8 T cells and *CXCR3* expressed by ASCs (P < 0.01 in 79/93 [84.9%] of samples) and found evidence for the same interaction (P<0.01 in 60/87 [69.0%] of samples) in B cells. In addition, we found weaker evidence for interaction between *TGFB1* expressed in NK cells and *CXCR4* on B cells [P<0.01 in 59/103 [59%] of samples), and for an interaction between *HMGB1* (from Tregs) and CXCR4. Interestingly, production of these ligands was generally promiscuous, suggesting that multiple cell types may contribute to a CSF milieu which favours B cell and ASC entry. Notably, these putative interactions were also observed in both the OIND and the ID cohorts, suggesting that these mechanisms are not specific to MS but rather may represent generic mechanisms for recruiting B cells and ASCs to CSF. To understand influences on B cell/ASC chemotaxis and survival beyond canonical chemokine interactions, we broadened our search to include any ligand-receptor pair. We considered interactions which were assessed in at least 90% of MS patients and, in those patients, achieved P<0.01 in at least 90%. Interestingly this approach pointed to a single ligand, *MIF* produced by CD4 and CD8+ T cells, acting via either a CD74-CXCR4 heterodimer on B cells, or via a CD44-CD74 heterodimer. We observed evidence for the MIF – CD74-CXCR4 interaction in the OIND B cells as well, again suggesting a general mechanism for B cell recruitment to the CNS rather than an MS-specific phenomenon [2]. A – correlation plot showing the Pearson's correlation coefficient for cell type proportions in MS CSF. Only pairwise correlation coefficients with a P value below the Bonferroni-adjusted threshold (alpha = 0.05) are shown. B – heatmap showing top prioritised chemokine – chemokine receptor interactions predicted to be active in MS CSF samples. The x axis shows the interaction, the y axis depicts the cell type, and the colour of the tile indicates the percentage of samples in which the interaction was predicted to be active (at a Bonferroni-adjusted P value threshold of alpha = 0.05). The left panel shows the 'target' of the interaction, i.e. the cell on which the receptor is expressed. C – UMAP plots showing B cells and ASCs from CSF and PBMC. The top panel shows the re-clustered B cells/ASCs with annotations from CellTypist. The panels below show expression of the chemokine receptors CXCR3 and CXCR4 in B cells/ASCs cells in CSF (left panels) and PBMC (right panels). IGHD and CD27 expression are also shown to highlight the continuum of B cell maturation in UMAP space.

Differential expression & pathway analysis

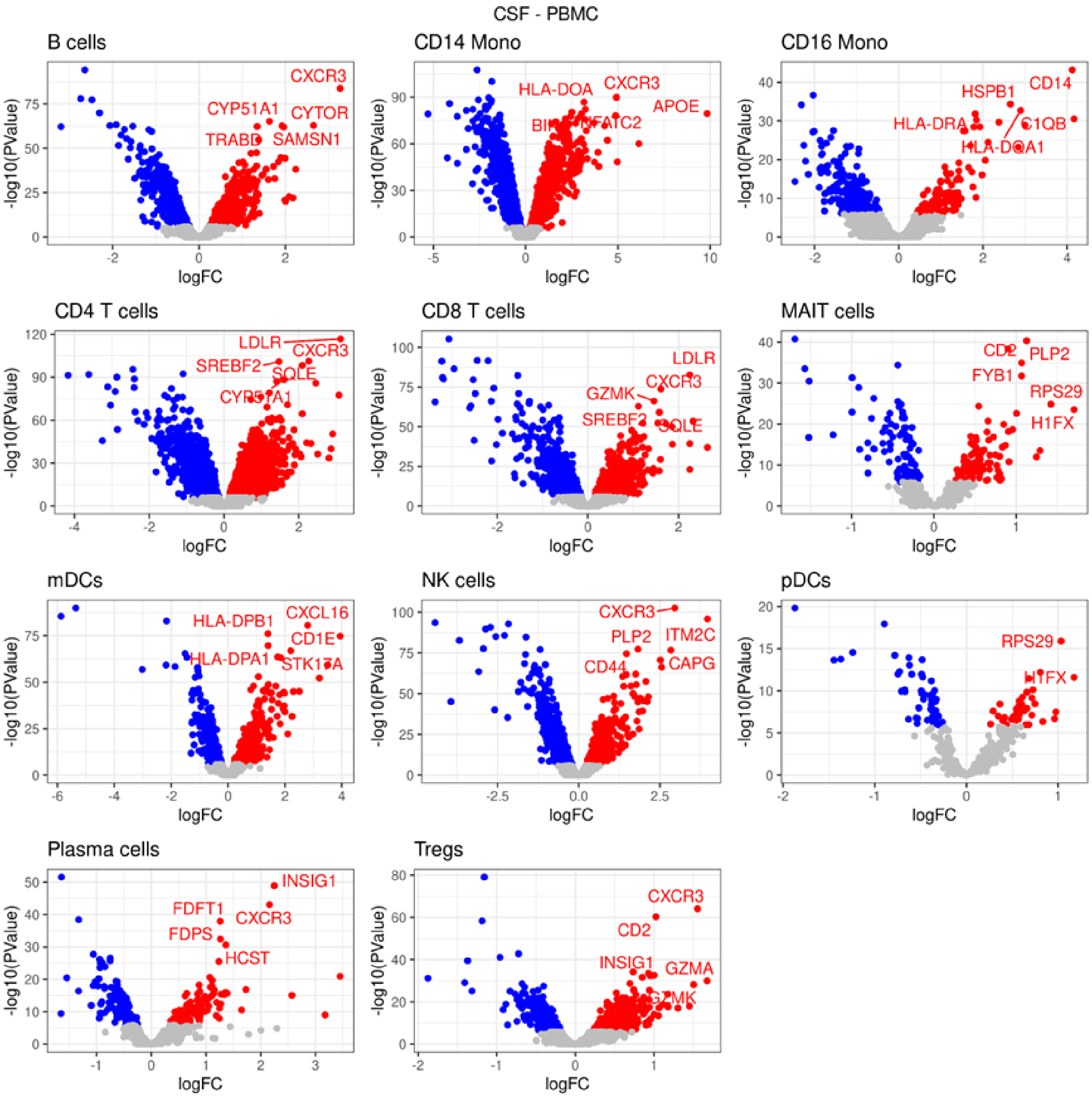

Supplementary figure 8: **Altered gene expression in CSF leukocytes, related to figure 2.** We first contrasted the transcriptional profiles of CSF leukocytes with PBMC leukocytes in a combined analysis, pooling samples across diseases. Differential expression testing revealed very few statistically significant differentially expressed genes between MS and controls in PBMCs. At a gene set level, this comparison revealed enrichment of genes involved in TNFa and NFkB signaling in the MS PBMCs, both pathways heavily implicated by GWAS findings. In PBMC, we observed strikingly few differences in gene expression between MS and either control cohort at an individual gene level. We found upregulation of the calcium-binding proteins *S100A8, S100A9, and S100A12* in MS CD4 T cells and of *S100A9* in MS CD8+ T cells compared with NIND PBMC. We also observed upregulation of *IL1B* - the gene for the interleukin 1-beta subunit - in both CD4+ and CD8+ T cells. There were very few significant differentially expressed genes contrasting MS PBMCs with either OIND or ID PBMCs, suggesting that these changes reflect non-specific CNS-directed immune responses rather than being MS-specific (supplementary table 6). Gene set enrichment analysis (GSEA) implicated multiple pathways as enriched in multiple cell types

from MS patients, including TNFα signalling via NFκB, inflammatory response genes, JAK-STAT signalling, and complement. These results suggest that the peripheral blood immune compartment in MS (and other CNS inflammatory states) shows transcriptional evidence of a pro-inflammatory phenotype, despite being compositionally similar to healthy controls. The image shows volcano plots displaying results of differential expression testing, comparing gene expression in each cell type between CSF and PBMC in a pooled analysis of all samples (i.e. disregarding disease phenotype). Each dot represents a gene tested, the y axis shows the -$\log_{10}$(P value), and the x axis shows the $\log_2$-fold change in transcript abundance. Genes coloured in red are up-regulated in CSF compared with PBMC. Genes coloured in blue are down-regulated in CSF compared with PBMC. Tests with a P value below the Bonferroni-corrected threshold of alpha = 0.01 are shown in grey.

B cell repertoire

	MS			OIND			ID		
**Ig gene family**	Direction	P	logFC	Direction	P	logFC	Direction	P	logFC
**IGHV4**	Up	<0.001	0.53	NS	0.773	-0.15	NS	0.968	0.02
**IGKV6**	Up	<0.001	1.44	NS	0.18	-1.81	NS	0.306	-0.67
**IGKV1**	Up	0.002	0.17	NS	0.743	-0.07	NS	0.03	-0.27
**IGKV3**	Up	0.003	0.20	NS	0.718	-0.08	NS	0.889	0.02
**IGKV2**	Up	0.015	0.29	NS	0.373	0.34	NS	0.693	0.09
**IGLV4**	NS	0.026	-0.61	NS	0.336	0.82	NS	0.542	0.25
**IGKV4**	NS	0.102	0.21	NS	0.135	0.62	NS	0.797	-0.07
**IGLV9**	NS	0.129	-0.60	NS	0.267	-1.45	Up	<0.001	2.47
**IGLV7**	NS	0.202	-0.36	NS	0.647	0.33	NS	0.372	-0.42
**IGKV5**	NS	0.523	0.19	NS	1	-0.01	NS	0.243	0.89
**IGHV6**	NS	0.552	0.26	NS	1	-0.04	Down	<0.001	-2.80
**IGHV3**	NS	0.682	-0.04	NS	0.23	0.40	NS	0.649	0.16
**IGLV8**	NS	0.699	-0.11	NS	0.259	1.09	NS	0.764	-0.18
**IGLV10**	NS	0.726	0.13	Up	0.006	3.03	NS	0.265	-0.82
**IGLV5**	NS	0.844	-0.08	NS	0.796	0.30	NS	0.478	-0.50
**IGLV6**	NS	0.975	0.01	NS	0.557	0.40	NS	0.373	0.46
**IGHV2**	Down	0.002	-0.97	NS	0.892	0.11	NS	0.257	-0.72
**IGHV7**	Down	0.003	-1.03	NS	1	-0.04	NS	0.207	0.52
**IGLV2**	Down	0.01	-0.27	NS	0.448	-0.27	NS	0.48	0.14
**IGHV1**	Down	0.013	-0.46	NS	0.303	-0.64	NS	0.702	-0.21
**IGHV5**	Down	<0.001	-0.91	NS	0.184	-1.01	NS	0.441	-0.46
**IGLV1**	Down	<0.001	-0.43	NS	0.594	-0.16	NS	0.773	-0.06
**IGLV3**	Down	<0.001	-0.41	NS	0.537	-0.22	NS	0.091	0.33

Supplementary table 11: preferential usage of specific Ig gene segments in CSF vs PBMC, related to figure 3. The table shows the results of differential abundance testing, comparing the relative usage of each specific gene segment in CSF vs PBMC cells within each disease cohort. The 'direction' column indicates whether the gene segment was used more or less in CSF than in PBMC for results with a False Discovery Rate of <10% - other results are shown as not significant (NS). LogFC refers to the $\log_2$ fold change of the proportion of cells expressing the gene segment in CSF vs PBMC.

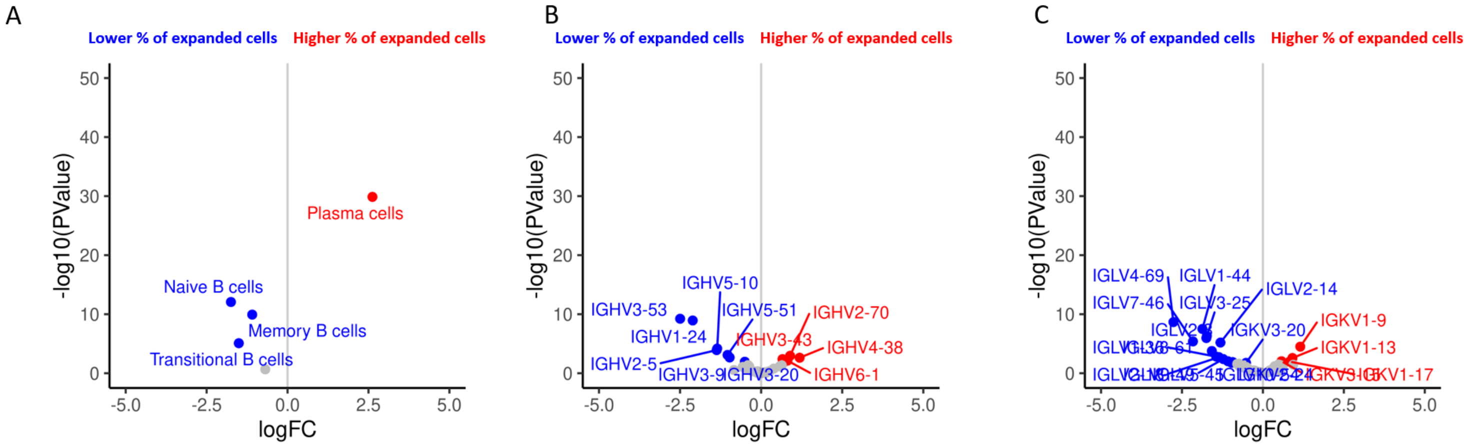

Supplementary figure 9: Characteristics of clonally-expanded B cells, related to figure 3. We compared the isotype usage and Ig gene usage of clonally-expanded cells vs non-expanded cells across all phenotypes in a pooled manner. These analyses demonstrated that clonal cells are largely IgG1+ ASCs cells with a bias towards specific IGHV genes. The image shows volcano plots comparing cell type proportions (A), IGHV (B) and IGKV/IGLV, (C) gene usage in expanded clones vs non-expanded cells.

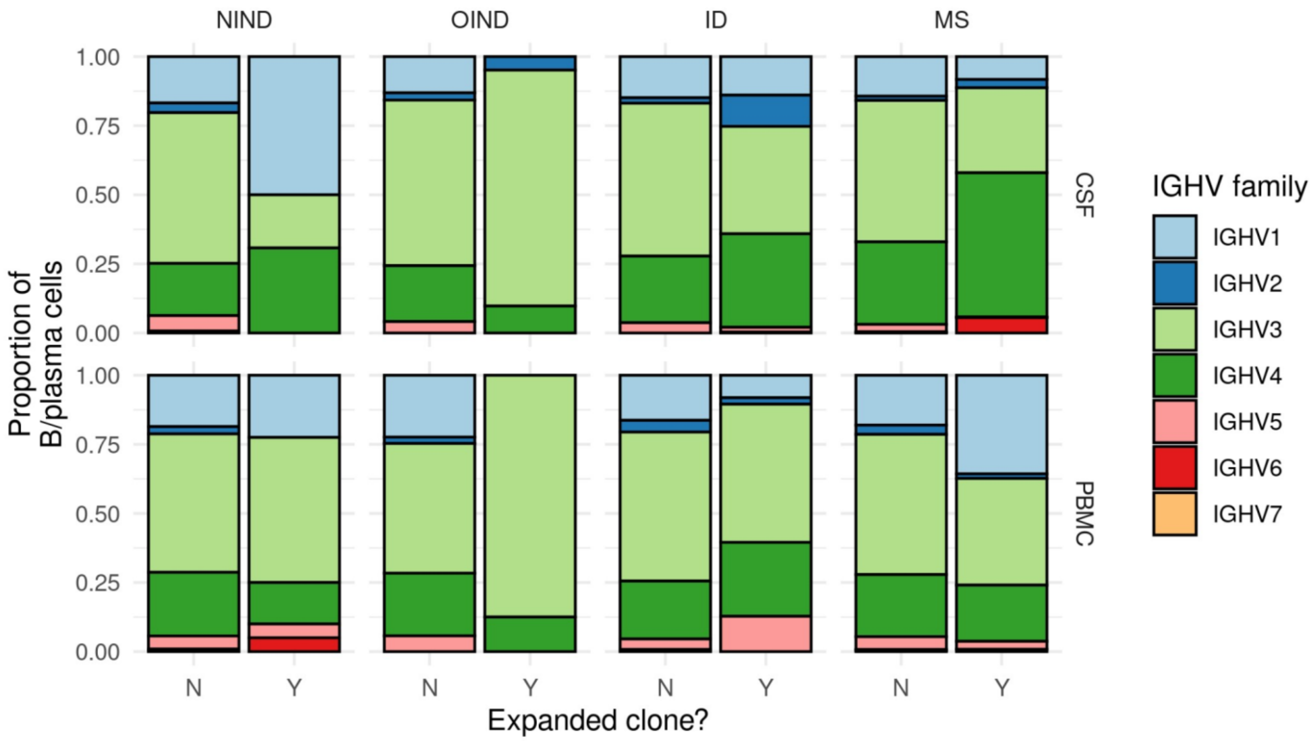

Supplementary figure 10: Immunoglobulin variable chain gene usage according to site, clonal status, and disease status, related to figure 3. Barplots showing bias towards IGHV4 usage in clonal B cells/ASCs is particularly prominent in the MS cohort.

	MS			ID		
Gene	logFC	P	FDR	logFC	P	FDR
CAPZB**	0.78	1.67E-06	2.27E-05	0.80	4.48E-05	0.01
SUB1**	1.25	4.80E-09	3.10E-07	0.93	2.91E-04	0.02
ARPC5**	0.74	1.11E-04	7.01E-04	0.89	7.84E-04	0.04
CCDC50*	0.90	3.31E-06	3.97E-05	0.50	0.03	0.26
CTSH*	0.74	1.36E-04	8.12E-04	0.44	0.06	0.39
IFI30*	0.89	2.57E-05	2.15E-04	0.66	0.07	0.41
TMSB4X*	0.85	1.22E-08	6.59E-07	0.42	0.08	0.41
ARPC1B	0.84	6.29E-08	2.26E-06	0.38	0.11	0.48
VOPP1	0.90	2.03E-07	4.38E-06	0.36	0.11	0.49
MT-ND5	0.73	2.17E-05	1.89E-04	0.35	0.12	0.49
EVI2B	0.68	2.60E-05	2.15E-04	0.27	0.15	0.51
LSP1	0.74	4.74E-07	9.01E-06	0.22	0.25	0.65
LBH	0.98	1.51E-07	4.38E-06	0.24	0.32	0.71
RAC2	0.50	3.45E-05	2.79E-04	0.16	0.40	0.77
HCLS1	0.64	1.34E-04	8.12E-04	0.14	0.45	0.80
CD53	0.64	1.64E-05	1.48E-04	0.15	0.53	0.85
IGKV3-15	2.35	1.05E-04	6.76E-04	0.10	0.90	0.99
TMBIM6	0.60	5.00E-05	3.84E-04	-0.01	0.97	1.00
HLA-DRA	0.50	9.93E-05	6.68E-04	0.00	0.99	1.00

Supplementary table 12: genes associated with clonal expansion of memory B cells, related to figure 3. The table shows the genes surpassing Bonferroni's adjusted alpha of 5% for the differential expression analysis of clonally-expanded memory B cells vs unexpanded cells in MS CSF. Only upregulated genes with a log-fold change of > 0.5 are shown. For each gene, the data from the ID cohort is also shown. ** indicates genes which also surpassed an FDR of 5% in the ID cohort. * indicates genes with a weakly suggestive effect in the same orientation in the ID cohort (at $P < 0.1$). A positive log-fold change indicates higher expression among expanded cells.

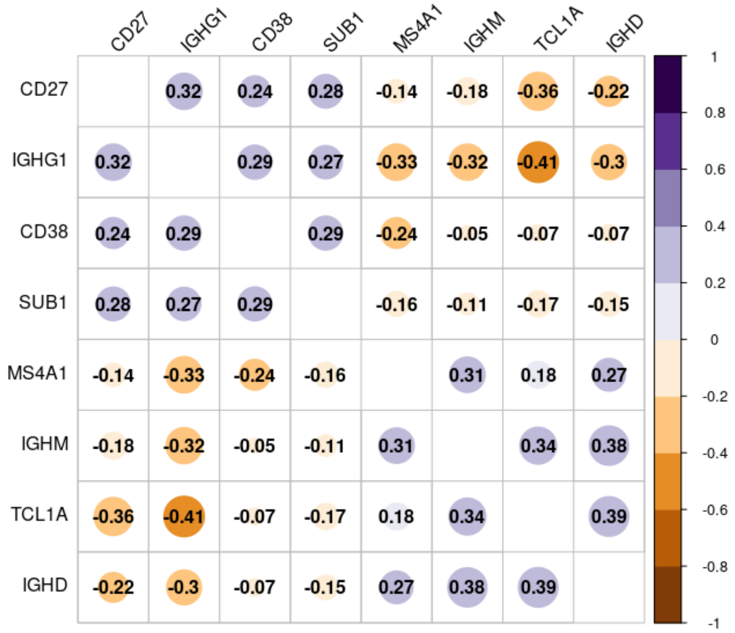

Supplementary figure 11: **SUB1 expression correlates with markers of B cell maturation, related to figure 3.** Correlation plot showing the spearman correlation between expression levels of *SUB1* and canonical markers of B cell differentiation. The numbers indicate the correlation coefficient. *SUB1* was positive correlated with CD27, IGHG1, and CD38 expression, and negatively correlated with CD20, IGHM, TCL1A, and IGHD (which delineate naïve B cells). The top clone-defining gene, *SUB1*, was highly expressed in memory B cells, ASCs, and the small number of germinal centre B cells. Expression of *SUB1* was highly correlated with markers of B cell differentiation, suggesting that the observed clonal signature is likely to reflect active B cell maturation into an ASC phenotype. We found several clonal families within which there was evidence of class-switching and/or multiple cell types. Most of these diverse clonal families consisted of both memory B cells and ASCs, suggesting synchronous generation of memory B cells and ASCs from germinal centre reactions. Broadly, isotype expression was similar for all clonal members, however we found several clones in which there was evidence of switching between IgD and IgM. We focused on clonal families displaying heterogeneity in isotype usage or cell type, as these provide a snapshot into a dynamic immune response. For instance, one clonal group identified in an MS patient consisted of germinal centre B cells, naïve B cells, and memory B cells that were present on both side of the blood-brain barrier. All of these cells expressed IgD/IgM. Intriguingly, despite having undergone extensive clonal expansion, the CDR3 sequences of these B cells remained germline, i.e. none of the cells displayed hallmarks of SHM. Overall, we found 81 such examples of expanded clonal families in which all cells had unmutated BCRs and expressed IgD/IgM. This phenomenon is not specific to MS, as we detected similar clones in all control cohorts (15 MS patients, 7 IDs, 1 OIND, and 3 NINDs). We found evidence of shared clones between individuals – we observed five ‘public’ clones in which B cells with near-identical BCRs were present in more than one patient. While two of these clones were MS-specific, the other three were not. This is consistent with published data suggesting that a large portion of the B cell repertoire is shared between individuals. To determine the antigen-specificity of the expanded clones we identified, we compared the CDR3 sequences of clonally-expanded B cells in MS patients in our dataset with those from a recently-published dataset of sorted ASCs from MS CSF[3]. Although we found no perfect matches, this was unlikely given that many clonally-expanded cells have undergone somatic hypermutation. We therefore calculated the length-normalised hamming distance, a measure of similarity, between all B cells in our dataset and the published work, restricting to only those cells with identical length CDR3s. We found 582 B cells which bore strong similarity to a published CDR3 amino acid sequence (length-normalised Hamming distance >70%). Of these 582 cells, we found two B cells with identical IGHV, IGL/KV, IGHD, IGHJ, and IGL/KJ gene usage to the corresponding B cell in the dataset from Steinman *et al*[3]. These cells were both IGKV3-expressing (*IGK3-20* and *IGKV3-66*), IGHV3-expressing (*IGHV3-7* and *IGHV3-66*) IgG1+ ASCs, both were present in the CSF of MS patients (one in each), and both had high similarity to the published sequence (Hamming distances of 90% and 88%). Interestingly, neither was part of an expanded clone. The antibody specificity of these CDR3 sequences is not clear, and further work is required to clarify whether these are MS-specific sequences or merely reflect public clones.

TCR repertoire

	EBV		CMV	
Phenotype	CSF	PBMC	CSF	PBMC
MS	39 / 118 (33.1%)	22 / 75 (29.3%)	49 / 118 (41.5%)	41 / 75 (54.7%)
ID	3 / 21 (14.3%)	2 / 4 (50%)	12 / 21 (57.1%)	3 / 4 (75%)
OIND	5 / 16 (31.2%)	6 / 11 (54.5%)	4 / 16 (25%)	5 / 11 (45.5%)
NIND	7 / 35 (20%)	8 / 28 (28.6%)	10 / 35 (28.6%)	16 / 28 (57.1%)

Supplementary table 13: viral-specific TCRβ chain frequency in CSF and PBMC, related to figure 4. Data show the number of patients from each group with at least one TCRβ predicted to bind to EBV / CMV epitopes. The denominator in each case is the number of patients with at least one T cell in the compartment. Pathogen-specific TCRs were defined as a perfect match between the TRBV gene and CDR3 amino acid sequence in the 5' single-cell dataset and the VDJDB reference dataset.

Expression QTLs

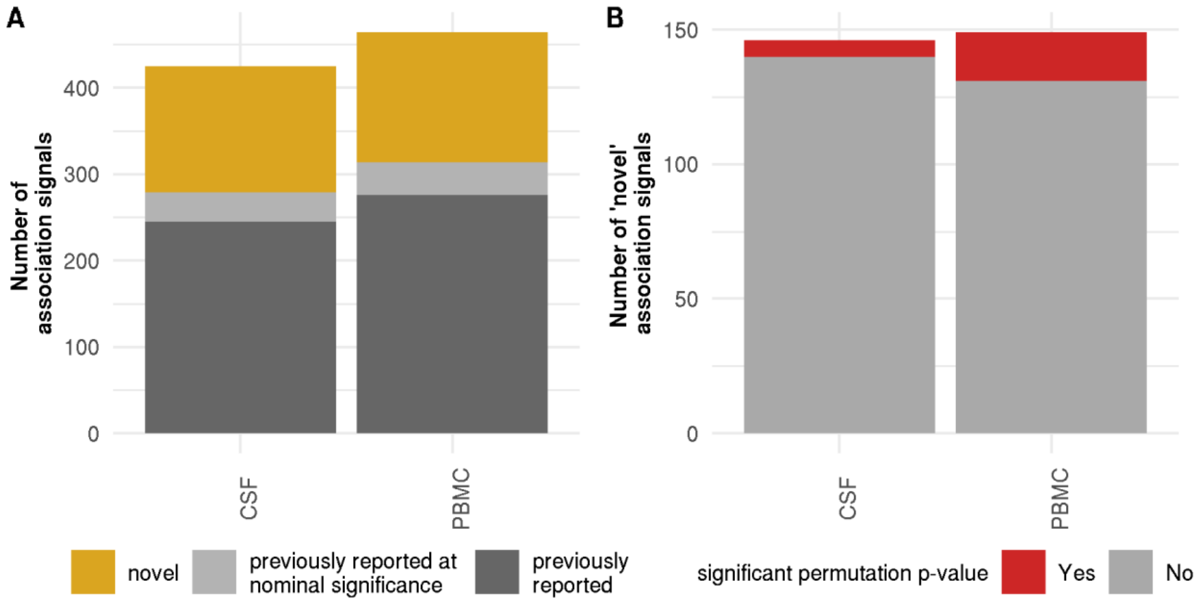

Supplementary figure 12: **expression QTLs in CSF and PBMC, related to figure 5.** A - Number of association signals (with FDR < 10%) that have been previously described by the Gtex or the eQTLGen consortium or Yazar et. al at significant or (only for eQTLGen) nominal significance level. B - Results of permutation analysis with a maximum of 1 million permutation for "novel" eQTL associations.

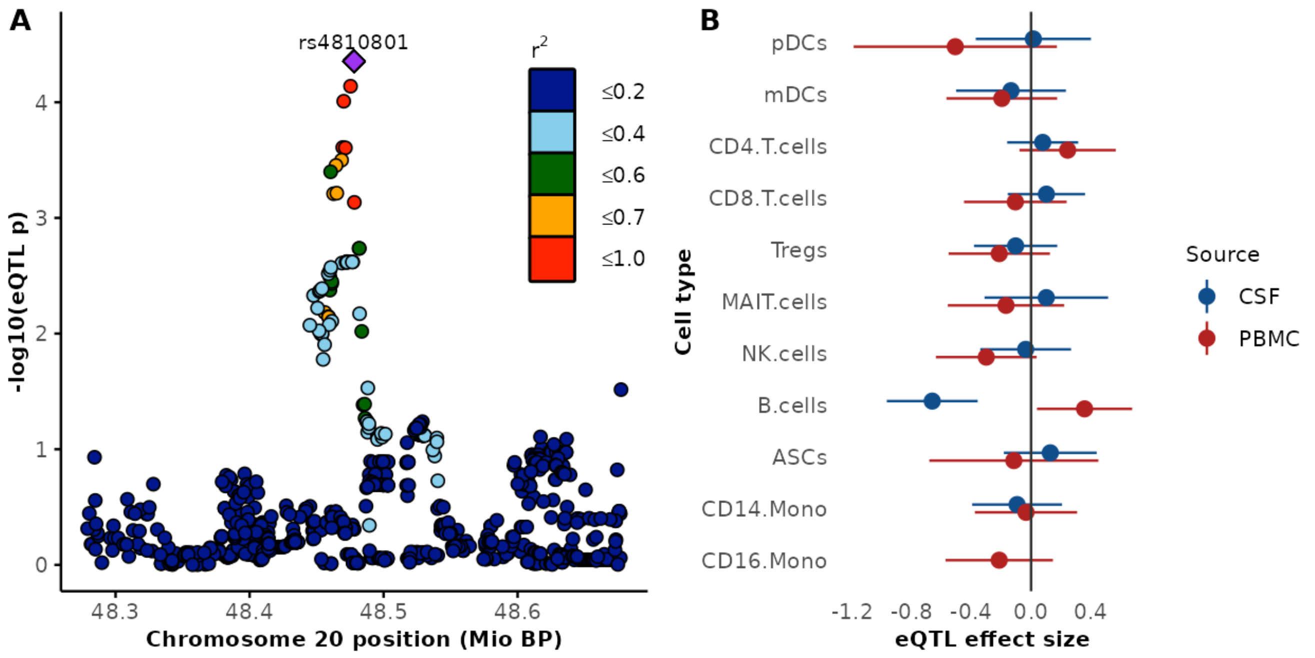

Supplementary figure 13: CSF B cell-specific eQTL for PREX1, related to figure 5. An eQTL for PREX1 in B cells appears to be CSF and B cell specific. A - Regional association plot for a locus on chromosome 20 associated with PREX1 expression CSF B cells. B - forest plot showing the eQTL effect estimates + 95% confidence intervals of rs4810801 on PREX1 expression in different cell types and compartments, suggesting a specific effect for CSF B cells. Abbreviations: ASCs, antibody secreting cells; BP, base pairs; eQTL, expression quantitative trait locus; GWAS, genome wide association study; Mio, million; Mono, monocytes.

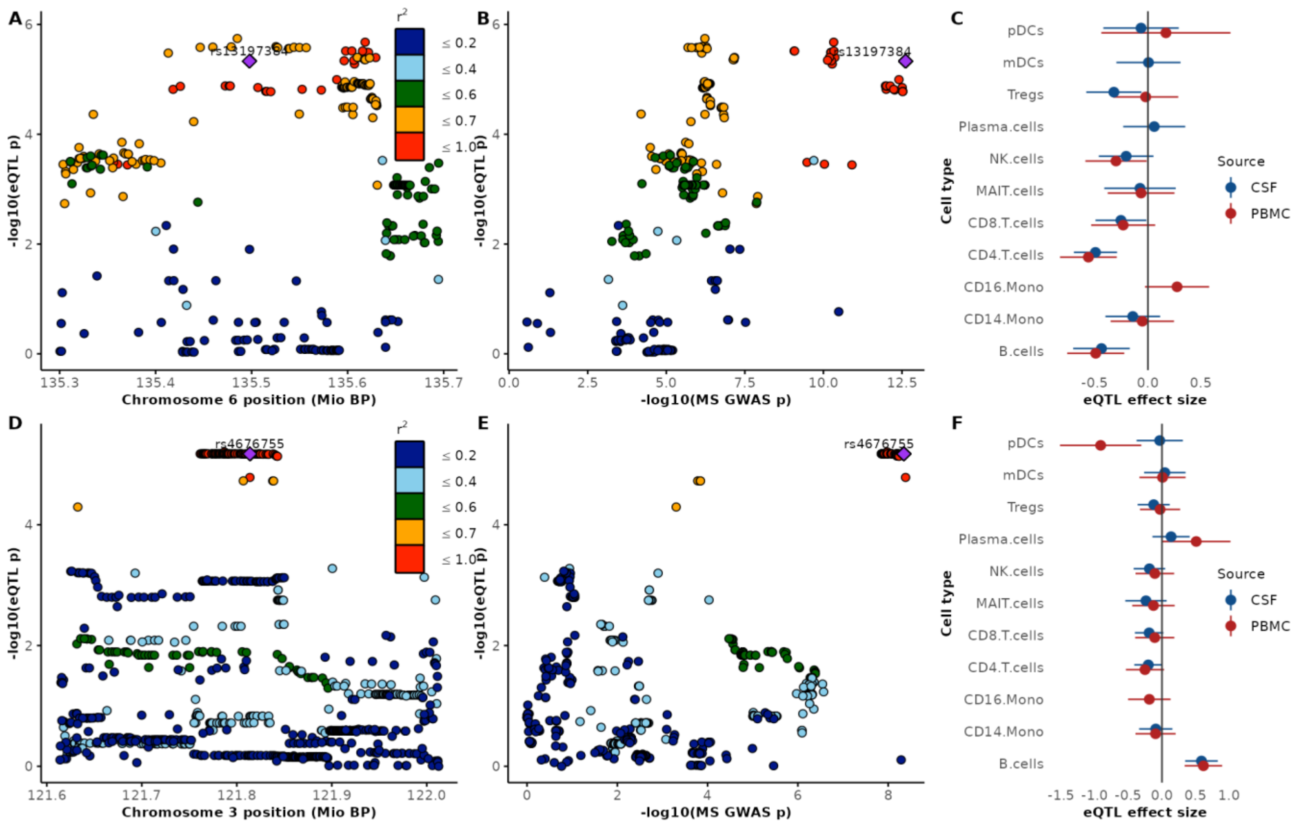

Supplementary figure 14: **Colocalisation between eQTLs and MS risk alleles, related to figure 5.** Replicated colocalizations between eQTLs for AHI1 and EAF2 in CSF cells and MS risk. A - Regional association plot for a locus on chromosome 6 associated with AHI1 expression CSF CD4+ T cells, B - correlation of eQTL p values and p values for MS risk (IMSGC 2019 susceptibility GWAS) for the same locus on chromosome 6, C - forest plot showing the eQTL effect estimates + 95% confidence intervals of rs13187384 on AHI1 expression in different cell types. D - Regional association plot for a locus on chromosome 3 associated with EAF2 expression CSF B cells. E - B - correlation of eQTL p values and p values for MS risk (IMSGC 2019 susceptibility GWAS) for the same locus on chromosome 3, F - forest plot showing the eQTL effect estimates + 95% confidence intervals of rs4676755 on EAF2 expression in different cell types, suggesting a B cell specific effect.

References

1. Dimitrov, D. *et al.* Comparison of methods and resources for cell-cell communication inference from single-cell RNA-Seq data. *Nat. Commun.* **13**, 3224 (2022).
2. Klasen, C. *et al.* MIF promotes B cell chemotaxis through the receptors CXCR4 and CD74 and ZAP-70 signaling. *J. Immunol.* **192**, 5273–5284 (2014).
3. Lanz, T. V. *et al.* Clonally Expanded B Cells in Multiple Sclerosis Bind EBV EBNA1 and GlialCAM. (2022).
